# Strategies of diaspore dispersal investment in Compositae: the case of the Andean highlands

**DOI:** 10.1093/aob/mcad099

**Published:** 2023-07-28

**Authors:** Carolina Tovar, Lucia Hudson, Francisco Cuesta, Rosa Isela Meneses, Priscilla Muriel, Oriane Hidalgo, Luis Palazzesi, Carlos Suarez Ballesteros, Eleanor Hammond Hunt, Mauricio Diazgranados, D J Nicholas Hind, Félix Forest, Stephan Halloy, Nikolay Aguirre, William J Baker, Stephan Beck, Julieta Carilla, Paúl Eguiguren, Elaine Françoso, Luis E Gámez, Ricardo Jaramillo, Luis Daniel Llambí, Olivier Maurin, Inga Melcher, Gemma Muller, Shyamali Roy, Paul Viñas, Karina Yager, Juan Viruel

**Affiliations:** Royal Botanic Gardens, Kew, Richmond, UK; Royal Botanic Gardens, Kew, Richmond, UK; Grupo de Investigación en Biodiversidad, Medio Ambiente y Salud – BIOMAS, Universidad de las Américas, Quito, Ecuador; Universidad Católica del Norte, San Pedro de Atacama, Chile; Herbario Nacional de Bolivia, Instituto de Ecología, Universidad Mayor de San Andrés, Bolivia; Laboratorio de Ecofisiología, Escuela de Ciencias Biológicas, Pontificia Universidad Católica de Ecuador, Quito, Ecuador; Royal Botanic Gardens, Kew, Richmond, UK; Institut Botànic de Barcelona (IBB, CSIC-Ajuntament de Barcelona), Barcelona, Catalonia, Spain; Museo Argentino de Ciencias Naturales (CONICET), Buenos Aires, Argentina; Banco de semillas, Jardín Botánico de Bogotá, Bogotá, Colombia; Royal Botanic Gardens, Kew, Richmond, UK; Royal Botanic Gardens, Kew, Richmond, UK; Royal Botanic Gardens, Kew, Richmond, UK; Royal Botanic Gardens, Kew, Richmond, UK; Biosecurity New Zealand, Ministry for Primary Industries, Wellington, New Zealand; Centro de Investigaciones Tropicales del Ambiente y Biodiversidad, Carrera de Ingeniería Forestal, Universidad Nacional de Loja, Loja, Ecuador; Royal Botanic Gardens, Kew, Richmond, UK; Herbario Nacional de Bolivia, Instituto de Ecología, Universidad Mayor de San Andrés, Bolivia; Instituto de Ecología Regional (IER), Universidad Nacional de Tucumán (UNT), Consejo Nacional de Investigaciones Científicas y Técnicas (CONICET), Tucumán, Argentina; Centro de Investigaciones Tropicales del Ambiente y Biodiversidad, Carrera de Ingeniería Forestal, Universidad Nacional de Loja, Loja, Ecuador; Royal Botanic Gardens, Kew, Richmond, UK; Laboratorio de Dendrología, Facultad de Ciencias Forestales y Ambientales, Universidad de Los Andes, Mérida, Venezuela; Laboratorio de Ecofisiología, Escuela de Ciencias Biológicas, Pontificia Universidad Católica de Ecuador, Quito, Ecuador; Instituto de Ciencias Ambientales y Ecologicas, Universidad de Los Andes, Mérida 5101, Venezuela; Consorcio para el Desarrollo Sostenible de la Ecorregión Andina, Germán Alemán E12-123, Quito, Ecuador; Royal Botanic Gardens, Kew, Richmond, UK; Institute for Biodiversity & Ecosystem Dynamics (IBED), University of Amsterdam, Amsterdam, The Netherlands; Naturalis Biodiversity Center, Leiden, The Netherlands; Royal Botanic Gardens, Kew, Richmond, UK; Royal Botanic Gardens, Kew, Richmond, UK; Fondo del Agua Quiroz, Piura, Perú; School of Marine and Atmospheric Sciences, Stony Brook University, Stony Brook, NY, USA; Royal Botanic Gardens, Kew, Richmond, UK

**Keywords:** Asteraceae, alpine ecosystems, Compositae, tropical Andes, diaspore morphological traits, diaspore investment

## Abstract

**Background and Aims:**

Understanding diaspore morphology and how much a species invests on dispersal appendages is key for improving our knowledge of dispersal in fragmented habitats. We investigate diaspore morphological traits in high-Andean Compositae and their main abiotic and biotic drivers and test whether they play a role in species distribution patterns across the naturally fragmented high-Andean grasslands.

**Methods:**

We collected diaspore trait data for 125 Compositae species across 47 tropical high-Andean summits, focusing on achene length and pappus-to-achene length ratio, with the latter as a proxy of dispersal investment. We analysed the role of abiotic (temperature, elevation and latitude) and biotic factors (phylogenetic signal and differences between tribes) on diaspore traits and whether they are related to distribution patterns across the Andes, using phylogenomics, distribution modelling and community ecology analyses.

**Key Results:**

Seventy-five percent of the studied species show small achenes (length <3.3 mm) and 67% have high dispersal investment (pappus length at least two times the achene length). Dispersal investment increases with elevation, possibly to compensate for lower air density, and achene length increases towards the equator, where non-seasonal climate prevails. Diaspore traits show significant phylogenetic signal, and higher dispersal investment is observed in Gnaphalieae, Astereae and Senecioneae, which together represent 72% of our species. High-Andean-restricted species found across the tropical Andes have, on average, the pappus four times longer than the achene, a significantly higher dispersal investment than species present only in the northern Andes or only in the central Andes.

**Conclusions:**

Small achenes and high diaspore dispersal investment dominate among high-Andean Compositae, traits typical of mostly three tribes of African origin; but traits are also correlated with the environmental gradients within the high-Andean grasslands. Our results also suggest that diaspore dispersal investment is likely to shape species distribution patterns in naturally fragmented habitats.

## INTRODUCTION

Dispersal investment, defined as how much the seeds of a plant invest in dispersal structures, is positively related to dispersal distances and is therefore a key element of the maintenance of species populations (Thomson *et al.*, 2018). Given that dispersal investment compares size/mass of diaspore appendages with that of the seed, comprehensive diaspore morphology data are fundamental to understand it. However, these data are scarce in fragmented regions, such as alpine habitats, where plant dispersal is more challenging. Alpine habitats are naturally fragmented owing to orographic and elevation gradients, but the levels of fragmentation and connectivity have changed over geological time. For example, upper forest lines shifted downwards during glacial periods, facilitating connectivity of alpine habitats, and vice versa during interglacial periods (Hooghiemstra and van der Hammen, 2004). Shifts in connectivity/isolation of alpine habitats have played a pivotal role in shaping species diversification owing to intermittent gene flow followed by allopatric isolation in these regions (e.g. Nevado *et al.*, 2018). Questions arise regarding the extent of diaspore dispersal investment in these dynamically fragmented habitats and what ecological and evolutionary forces shape it.

The Andes of South America are the longest mountain chain on Earth (>6500 km), with alpine habitats located approximately above 3200–3500 m a.s.l. in their tropical section, depending on latitude. Dispersal-related traits of tropical alpine species have scarcely been investigated (e.g. Tovar *et al.*, 2020; Llambí *et al.*, 2021), and very few studies have characterized the morphology of their diaspores (e.g. Melcher *et al.*, 2000, 2004). Compositae are among the most species-rich families in the high Andes and are also among the best-represented families in other open environments, such as deserts and steppes (e.g. Körner, 2003; Palazzesi *et al.*, 2022*a*: fig. S1). This success is attributed, in part, to the pappus, which is a modified calyx consisting of bristles, dry scales and/or awns that aid dispersal and is present in most Compositae (Kadereit and Jeffrey, 2007 [2006]; Funk *et al.*, 2009; Panero and Crozier, 2016). All these traits and their geographical abundance make Compositae an excellent case study to investigate diaspore morphological variation in the context of species ecology, diversification and distribution ranges in the Andes.

In Compositae, both the pappus and the achene generally constitute the diaspore, the reproductive portion of the plant that is dispersed. The development of appendages aiding dispersal increases the likelihood of reaching longer dispersal distances, and this dispersal investment seems to be a better predictor of dispersal distances than seed mass (Thomson *et al.*, 2018). However, the best model predicting dispersal distances includes both dispersal investment and plant height (Thomson *et al.*, 2018). On the contrary, bigger seeds/achenes might result in more competitive seedlings, owing to potentially more nutritional reserves available for their establishment, and higher seedling survival in harsh or variable environments (Meyer and Carlson, 2001; Moles, 2005). However, nutritional reserves will also be influenced by seed coat thickness (Chen *et al.*, 2020). Thus, different diaspore morphologies can provide different ecological strategies. It is important to determine the diversity of this morphology in high-elevation Andean Compositae living in highly fragmented habitats. This is particularly relevant because it has been suggested that species from oceanic islands and sky islands (e.g. the high Andes) tend to lose dispersal ability (Carlquist, 1966), although this hypothesis has been challenged recently (García-Verdugo *et al.*, 2017).

**Fig. 1.**
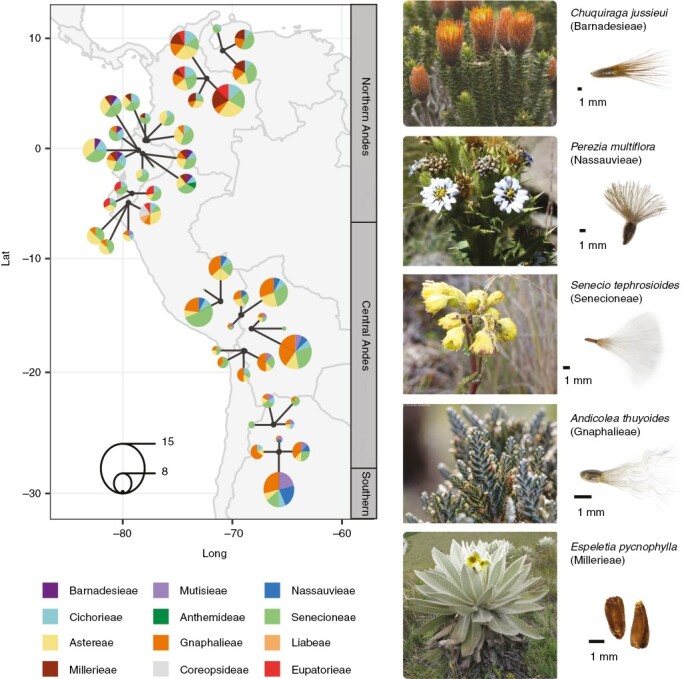
Study area and examples of Andean Compositae species and their diaspores. On the map on the left, the black dots show the approximate middle point location of each site, which is composed of three or four summits. Circle size represents the number of Compositae species in each summit. The tribe key is organized in rows, going from the top left to the bottom right, by the age of the split from their sister lineage, following Mandel *et al.* (2019), from older to younger estimate of stem node ages (see Fig. 2). Credits of photographs, from top to bottom: Ricardo Jaramillo, Ricardo Grau, Mauricio Diazgranados, Ricardo Jaramillo and Mauricio Diazgranados.

Diaspore morphology and thus, dispersal ability, directly affects how efficiently plant species persist in suitable areas or colonize them (Saatkamp *et al.*, 2019) and could facilitate migration through geographical barriers. Two main geographical and climatic barriers divide the Andean Cordilleras into the northern, central and southern Andes (Fig. 1). The Huancabamba depression, in the northern Cordilleras of Peru (~6°S), divides the northern Andes from the central Andes and is one of the lowest points in the tropical Andes (~2141 m a.s.l.). Between the central Andes and the southern Andes (~29°S) the Cordilleras narrow; elevation remains high up to 35°S, after which it decreases gradually, whereas rainfall increases gradually and substantially, especially on the western side of the Cordilleras, south of 36°S (Luebert and Pliscoff, 2006; Luebert and Weigend, 2014). This combination of climatic and geographical variation could have acted as barriers for some plant species (Luebert and Weigend, 2014), depending on their dispersal ability.

Environmental gradients, which are stronger over short distances in mountain regions, can shape dispersal-related traits. For example, dispersal modes are filtered along the minimum temperature gradient in the high tropical Andes, with endozoochorous species being more prevalent in areas with higher minimum temperatures and barochorous species in areas with lower minimum temperatures (Tovar *et al.*, 2020). Global patterns of diaspore size show that larger seeds are usually found closer to the equator (Moles *et al.*, 2007). This could potentially be attributable to higher precipitation and more constant environmental conditions found closer to the equator. Indeed, a modelling study showed that large seeds (or both small and large) can be optimal under high precipitation; however, the study also found that regions with constantly low precipitation are most likely to select for large seeds (Volis and Bohrer, 2013).

Along an elevation gradient, the lower air pressure found at higher elevations could hinder wind dispersal (Halloy, 1985, 1989), because objects will fall faster in less dense air, owing to reduced friction countering gravitational force and reduced buoyancy. Therefore, wind-dispersed species would need to compensate for reduced air density by increasing their dispersal aids at high elevations. Nevertheless, greater investment in larger appendages is energetically more costly (Bonte *et al.*, 2012). Hypotheses about changes in diaspore traits along environmental gradients have not yet been assessed in tropical mountain regions, such as the high Andes.

The evolutionary history of the Compositae lineages could also influence diaspore morphology. The family is likely to have originated in western Gondwana (i.e. southern South America, Antarctica) ~83 (95% CI, 64–91) Mya (Mandel *et al.*, 2019; Palazzesi *et al.*, 2022*b*). Several of the Compositae tribes originated and diversified in the New World (e.g. Barnadesioideae and Mutisioideae), although the origin of the lineages that currently have the largest number of species was estimated to have occurred in the Old World (Panero and Crozier, 2016). A major evolutionary radiation occurred in Africa between the Late Eocene and Early Oligocene (~36 Mya), and several of these lineages recolonized South America, such as Senecioneae and Gnaphalieae. Mandel *et al.* (2019) inferred further long-distance dispersal events from Africa to South and Central America in the late Oligocene–Miocene (~23 Mya), which gave rise to the Heliantheae tribal alliance, many of which subsequently diverged within South America, such as Millerieae, Eupatorieae and Coreopsideae (Panero and Crozier, 2016). Therefore, the evolutionary origin of diaspore traits in each lineage could have occurred earlier than the colonization of their current habitats.

Using Compositae species recorded in 47 plant communities from mountain summits located across the high tropical Andes and monitored by the GLORIA (Global Observation Research Initiative in Alpine Environments) Andes network (Cuesta *et al.*, 2017), we seek to answer the following three questions. First, what are the most common diaspore trait values among high-Andean Compositae? Second, what abiotic and biotic factors influence the observed diversity and distribution of diaspore traits? Third, are diaspore traits related to the ability of high-Andean Compositae to cross geographical barriers and thus affect their distribution patterns across the Andes? We focus on two key traits linked to dispersal in Compositae, achene length and pappus-to-achene length ratio, the latter a proxy of dispersal investment. We first hypothesized that most high-Andean Compositae will have small achenes and invest little in developing large appendages to minimize costs owing to cold temperatures (H1). Secondly, we predict that abiotic conditions shape diaspore morphology within the alpine habitat: larger achenes will be found closer to the equator (Moles *et al.*, 2007), and higher dispersal investment will be found at higher elevation (H2), compensating for the decrease in air density (Halloy, 1985). Thirdly, we hypothesize that diaspore traits will show phylogenetic signal and thus vary between tribes (H3). Lastly, we hypothesize that species restricted to the high Andes will tend to favour low dispersal investment and thus have low ability to cross barriers (H4) because, in sky islands, the presence of a hostile surrounding matrix would have negatively selected individuals with long dispersal strategies (Carlquist, 1966).

## MATERIALS AND METHODS

### Study area and plant community data

Our study area encompasses 13 sites along the high tropical Andes (>3200 m a.s.l.) spanning 4200 km from Venezuela to Argentina (Cuesta *et al.*, 2017). It covers the grassland landscapes, interspersed by shrubs, of the northern Andes (Páramo, 11°N–7°S) and the grasslands of the central Andes (Puna, from 7 to 26–29°S). At each site, the GLORIA-Andes network (Cuesta *et al.*, 2017) has set up permanent vegetation plots (8–16 plots, each measuring 1 m^2^) on three or four different summits for long-term monitoring. Here, we used the vegetation composition recorded between 2012 and 2013 for a total of 49 summits. We investigated the diaspore morphology of all 125 Compositae species recorded on 47 of these summits (Compositae species were absent in the plots from two summits; Fig. 1; Supplementary Data Table S1). The most species-rich genus in the studied area was *Senecio* (19 spp.), followed by *Baccharis* and *Rockhausenia* (eight spp. each).

### Data collection

#### Diaspore trait data.

We characterized diaspore morphology by collecting data on achene length (*n* = 122 spp.), achene width (*n* = 104 spp.) and pappus length (*n* = 125 spp.) from herbarium specimens, scientific articles and books (Supplementary Data Methods S1). Previous studies have used the ratio of pappus to achene volume or mass as a proxy for estimating dispersal investment (Cody and Overton, 1996; Thomson *et al.*, 2018). In the absence of volume or weight data, we used pappus-to-achene length ratio (hereafter, dispersal investment). A value of one indicates that the achene and the pappus have the same length; two indicates that pappus length is double that of the achene, and so on. For all further analyses we used two traits: achene length and dispersal investment. We also downloaded achene length data for other Compositae species across the world from available online databases (*n* = 155) to compare them with our results (Supplementary Data Methods S1).

Appendages were classified into five categories following Roque *et al.* (2009): (1) bristles; (2) awns; (3) scales; (4) winged; and (5) epappose (no dispersal appendage), based on written descriptions and herbarium specimens. Only three of the species have awns (*Bidens andicola* Kunth, *Coreopsis venusta* Kunth and *Stevia andina* B.L.Rob.), for which the dispersal investment metric will not be useful because they are dispersed by animals rather than by wind; however, this had little impact in the overall analyses. Species trait values were used to study variation in diaspore morphology. Mean values of achene length and dispersal investment were estimated for each genus to represent species without data. This full dataset (including traits at the genus level) was used to estimate community median trait values per summit.

#### DNA sequencing and phylogenomic analysis.

Total DNA was extracted successfully from leaf tissue samples of herbarium specimens for 81 species (Supplementary Data Table S2) using a modified CTAB protocol for herbarium specimens (Doyle and Doyle, 1987). We generated target capture data using the Angiosperms353 kit developed to retrieve 353 nuclear genes across angiosperms (Johnson *et al.*, 2019; Baker *et al.*, 2022). Genomic libraries were constructed as optimized by Viruel *et al.* (2019) for half volumes of the NEBNext® Ultra™ II DNA Library Prep Kit for Illumina® (New England Biolabs, Ipswich, MA, USA) and purified using AMPure XP magnetic beads and multiplexed using NEBNext® Multiplex Oligos for Illumina® (Dual Index Primer Sets I and II). Pools containing 13 genomic libraries mixed in equimolar conditions were subsequently enriched with half reactions of the Angiosperms353 probe kit following myBaits® kit manual v.3.02 (Arbor Biosciences). The complete and detailed protocols are described by Brewer *et al.* (2019). The DNA concentration and library quality were calculated using a Quantus™ fluorometer (Promega Corp.) and an Agilent 4200 TapeStation (Agilent Technologies, Santa Clara, CA, USA), respectively. Sequencing was performed on a HiSeq (Illumina, Inc.) by Macrogen (Seoul, South Korea) producing 2 × 150 bp paired-end reads. Raw reads were submitted to the European Nucleotide Archive under umbrella project PRJEB35285.

We used Trimmomatic v.0.35 (Bolger *et al.*, 2014) to discard low-quality reads and trim adapters based on the reports generated by FastQC v.0.11.7 (Andrews, 2010). We used HybPiper v.1.3.1 (Johnson *et al.*, 2016) to retrieve the 353 nuclear loci using a combination of map to reference and *de novo* assembly methods for all samples.

Alignments were generated in MAFFT 7.305b (Katoh, 2002) using the command *auto*, then edited with trimAL v.1.4.rev22 (Capella-Gutierrez *et al.*, 2009) using the *automated1* parameter. Eleven genes having >50 % of missing taxa were discarded. The number of gene sequences missing per species in the final alignments was 19.5 on average, ranging from 4 to 53, except for *Monticalia vaccinioides* (Kunth) C.Jeffrey, with missing data for 156 genes. There was 13.45% missing data overall in the alignments. Phylogenetic analyses were conducted for the concatenated and partitioned dataset of nuclear data (i.e. supermatrix approach) and by estimating a species tree from individual phylogenetic trees reconstructed for each nuclear locus independently (i.e. multispecies coalescent approach). Phylogenetic trees were reconstructed using RAxML-NG (Kozlov *et al.*, 2019) and IQ-TREE (Nguyen *et al.*, 2015), with the GTR+GAMMA (Yang, 1994) substitution model and 1000 bootstrap replicates. Concatenated datasets were built with FASconCAT-G v.1.04 (Kück and Longo, 2014). We used ASTRAL‐III (Zhang *et al.*, 2018) to construct a species tree based on the independent nuclear gene trees. We also used SVDquartets analysis as implemented in Paup* v.4.0a146 (Swofford, 2003) to evaluate 10 000 000 random quartets (or all possible quartets, if lower than this) and calculated bootstrap support by running 10 000 bootstrap replicates. We used treePL (Smith and O’Meara, 2012) to estimate divergence times using the fossil *Mutisiapollis telleriae* found with macrofossil *Raiguenrayun cura* and dated to 47.5 (50.2–60.0) Mya, both recovered from Patagonian deposits (Barreda *et al.*, 2012), as a calibration point at the split between subfamilies Mutisioideae and Asteroideae. Following Panero and Crozier (2016) and Mandel *et al.* (2019), we applied a minimum age of 50.16 Mya and a maximum value of 60.00 Mya to the node connecting *Perezia* and *Hieracium* clades. The analysis was run in two consecutive steps: (1) using the ‘prime’ option to select the most optimal parameter values; and (2) a ‘thorough’ analysis by applying opt = 2, optad = 2 and optcvad = 2.

#### Species occurrences.

A total of 5322 georeferenced occurrence records for the 125 Compositae species (mean = 42 per species) were used to model their species distribution range. These were obtained from an updated version of the occurrence data used by Cuesta *et al.* (2020) that includes data from GBIF (https://www.gbif.org/), Tropicos (https://www.tropicos.org) and South American herbarium specimens (LPB and MERF).

### Data analyses

#### Variation in diaspore morphology.

We estimated ranges for achene length, achene width, pappus length and dispersal investment. We plotted achene length vs. dispersal investment to explore how these two traits covary and, for achene length, we used a Wilcoxon rank sum test to compare the lengths between our dataset and that from other Compositae collected from online available datasets.

#### Diaspore morphological traits across environmental gradients.

We tested whether achene length and dispersal investment were associated with environmental conditions using a fourth-corner analysis (Dray and Legendre, 2008). The analysis combines three matrices [R (summit × environmental variables), L (summits × species) and Q (species × traits)] using G statistics to test for correlation, assessed via 3000 permutations of species data within each row of matrix L (model 3) (Legendre and Legendre, 2012). We used the community data of each summit to build the L matrix, and the minimum temperature (bio6 from WorldClim; Hijmans *et al.*, 2005), elevation and distance from the equator (measured in degrees) of each community to build the R matrix. We used minimum temperature because this is known to be a limiting variable in alpine environments (Rundell *et al.*, 1994; Cuesta *et al.*, 2017), and in the high Andes it has been related to the filtering of dispersal mode and growth form (Tovar *et al.*, 2020; Llambí *et al.*, 2022). As elevation increases, air density decreases, and this will affect wind dispersal because terminal velocity will be higher (Liu *et al.*, 2021). We also included distance from the equator amongst our environmental variables because seasonality increases along this gradient (Körner, 2012). Precipitation data for the Andes from global datasets are less reliable; therefore, we did not include these data (Hijmans *et al.*, 2005).

#### Phylogenetic signal and variation in diaspore morphology across the phylogeny.

Phylogenetic signal was tested for diaspore traits and range size using the 81 species for which DNA sequencing was successful. We used Pagel’s *λ* (Pagel, 1999), which compares the phylogenetic signal with the Brownian motion (BM) model of trait change using the function fitContinuous from the R package geiger (Harmon *et al.*, 2008). Values of *λ* close to zero represent no signal, and values of one represent what you would expect with BM. We used a likelihood-ratio test to compare the model fitting of each trait with a BM model and with a phylogeny lacking information on evolutionary relationships (star phylogenetic tree). Significance was estimated assuming a χ^2^ distribution (Cadotte and Davies, 2016).

We used the most comprehensive phylogenomic and biogeographical study for Compositae as a reference to classify tribes according to their divergence times (Mandel *et al.*, 2019). First, we used the divergence times estimated for the stem nodes of each tribe to assign them to a geological epoch (Palaeocene, Eocene, Oligocene or Miocene) in which they originated. Second, we grouped tribes according to their biogeographical origin: (1) ‘pre-Andean’ (the stem node of the tribe originated in South America before the Oligocene Andean uplift); (2) ‘African’ (the stem node of the tribe is reconstructed as in Africa, and the lineage subsequently dispersed to South America); and (3) ‘Andean’ (the stem node of the tribe originated in the New World after the Andean uplift). We then compared trait values across tribes and within groups according to geological time and origin as described above.

#### Species distributions vs. diaspore strategies.

The distribution of species with at least ten georeferenced occurrences (100 species) was modelled using BIOMOD2 (Thuiller *et al.*, 2009), and the geographical coordinates of occurrences were used to extract data from the following WorldClim 30″ resolution variables: mean annual temperature, mean temperature diurnal range, total annual precipitation and precipitation of the coldest month (Hijmans *et al.*, 2005). Slope was derived from a digital elevation model at 15″ resolution from HydroSHEDS (https://www.hydrosheds.org/). We calibrated the models using 80% of the data and tested with the other 20% in five repetitions using three different algorithms, namely generalized additive model, random forest and MAXENT implemented in BIOMOD. Final maps were obtained using an ensemble model (probability weighted mean) using models whose true skill statistic and area under the curve evaluation metrics were >0.7 and >0.8, respectively (Liu *et al.*, 2011). Using expert knowledge, country flora catalogues and distribution maps from Plants of the World Online (http://powo.science.kew.org/), we trimmed the model outputs where distributions were overpredicted to obtain final potential distribution maps. For species with fewer than ten records (25 species) it was not possible to create a model; however, we wanted to represent these small ranges, mostly restricted to mountains, as accurately as possible. Therefore, circles of 2 km radius were created around each occurrence point (buffers). We took this conservative approach because climatic conditions vary a lot in mountains owing to elevation; therefore, anything >2 km (two pixels) could misrepresent the niche of the species.

Species range sizes (in squared kilometres) were estimated from the trimmed modelled maps and the 2 km buffer maps using the R package geosphere (Hijmans *et al.*, 2019). We classified species as restricted to the high Andes (86 spp.) or non-restricted (39 spp.) and compared differences in diaspore traits between the two groups using a Wilcoxon rank sum test. We subdivided the species further based on whether they are distributed in the northern, central and/or southern Andes using the following categories (see limits in Fig. 1): north (*n* = 40), centre (*n* = 42); north and centre (*n* = 37); centre and south (*n* = 5); and north, centre and south (*n* = 1). Given the low number of species of the last two categories, we analysed differences in traits between only the first three categories using the Games-Howell test in the R package PMCMRplus (Pohlert, 2021). We also analysed whether species distribution range size was related to the environmental variables using the fourth-corner approach described above.

## RESULTS

### Diaspore morphology

**Fig. 2.**
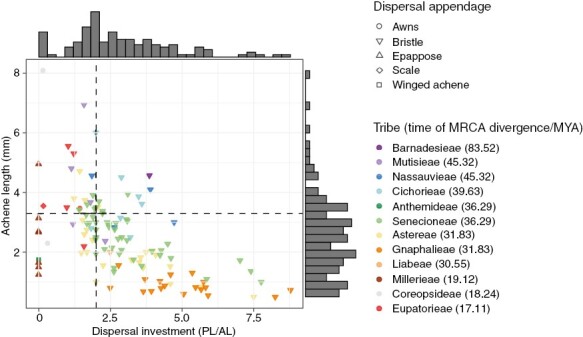
Variation in diaspore morphological traits across tribes of high-Andean Compositae. Dispersal investment [measured as pappus-to-achene length ratio (PL/AL)] vs. achene length. Vertical line marks a value of two for PL/AL, which indicates that the pappus is twice as long as the achene. Horizontal line shows the median value (3.3 mm) for achene length from other Compositae across the world (Supplementary Data Fig. S2). Tribes are ordered in the key from top to bottom, by time of divergence from their sister lineage following Mandel *et al.* (2019). Species are coloured by tribe, and shapes represent dispersal appendages. Abbreviation: MRCA, most recent common ancestor.

Achene length ranged between 0.5 and 8 mm (Supplementary Data Fig. S1), with *Bidens andicola*, which disperses by attaching to animal fur rather than by wind, presenting the highest value. Achene width ranged mostly between 0.16 and 2.4 mm, with one species, *Trichocline reptans* (Wedd.) Hieron., reaching 4.1 mm. In comparison to other Compositae species around the world (median = 3.3 mm), achenes measured in this study (median = 2.4 mm) were significantly smaller (*P* < 0.001; Supplementary Data Fig. S2). In fact, 75% of our species had achene length lower than the median value of other Compositae around the world. Pappus length ranged between 0.6 and 17 mm for pappose species. Dispersal investment varied from 0 (when pappus is absent) to 8.7 (Supplementary Data Fig. S1), with the latter value indicating that the pappus length was eight times longer than the achene length. Around 67% of the studied species had dispersal investment values higher than two, which indicated that the majority of species had pappus length at least twice as long the achene (Fig. 2). Species that had both small achenes (<3.3 mm) and low dispersal investment (less than two) represented 18% of the studied species.

The bristle pappus was the most common dispersal appendage among the studied species, with awns, scales, winged and epappose achenes making up only 8% of species (ten spp.) (Supplementary Data Table S1).

### Diaspore traits across environmental gradients

**Fig. 3.**
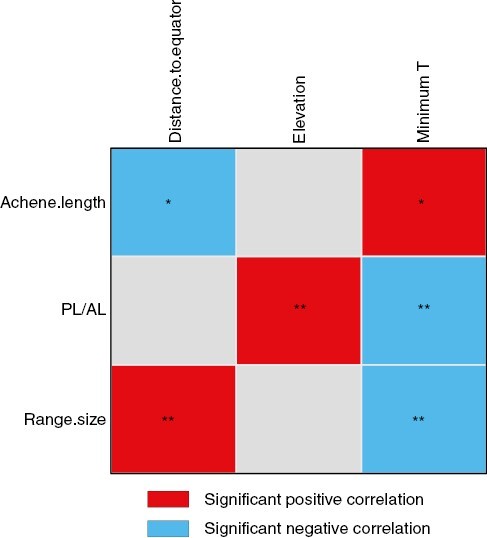
Results of the fourth-corner analysis that tested the association between diaspore morphological traits and range size vs. environmental variables. We tested the association between plant traits [achene length (AL), diaspore dispersal investment (pappus-to-achene length: PL/AL) and range size] and the following environmental variables: distance to the equator, elevation and minimum temperatures.

Achene length was negatively correlated with distance to the equator (*P* = 0.02) and positively correlated with minimum temperature (*P* = 0.02) (Fig. 3; Supplementary Data Table S3). Dispersal investment was positively correlated with elevation (*P* = 0.004) and negatively correlated with minimum temperature (*P* = 0.003). We also examined range size as a trait and found that larger range sizes were normally further from the equator and in areas with lower minimum temperatures (*P* = 0.003 in both cases).

### Phylogenomic inference

On average, we obtained 4 266 638 paired reads per sample, ranging from 396 960 to 17 001 680, from which 26.7% were on target on average, ranging from 12.3 to 54.5. These sequences allowed us to assemble 265 genes at 50% length on average, ranging from 90 to 330, relative to the full length of the Angiosperms353 reference target gene set, in total representing 71.4% on average of the total maximum length that could be recovered, ranging from 30.1 to 88.3. Sequencing results per sample are summarized in Supplementary Data Table S2.

Overall congruency was found between tree topologies reconstructed using supermatrix and multispecies coalescent approaches, and strong support values were obtained from the different analyses among clades (Supplementary Data Figs S3–S7). The splits between tribes were estimated to have taken place during the Eocene, but tribes Coreopsideae, Millerieae and Eupatorieae probably diverged from their sister lineage during the Oligocene. Crown node divergences for all tribes were estimated to have occurred during the Miocene.

### Diaspore traits across evolutionary history

**Fig. 4.**
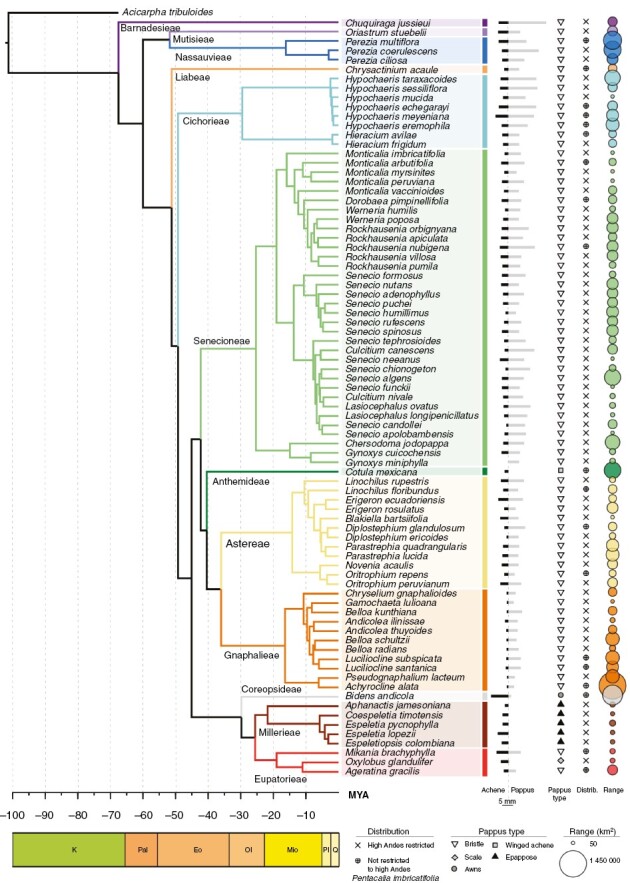
Phylogenetic tree for 81 of the 125 Compositae species studied here. To the right of the tree we present, for each species, the following: bars representing proportionally the achene (black) and pappus (grey) length, pappus type, whether the species is restricted to the high Andes or not, and coloured circles on the far right representing range size. Geological times are presented at the bottom, as follows: Eo, Eocene; K, Cretaceous; Mio, Miocene; Ol, Oligocene; Pal, Palaeocene; Pl, Pliocene; and Q, Quaternary.

**Fig. 5.**
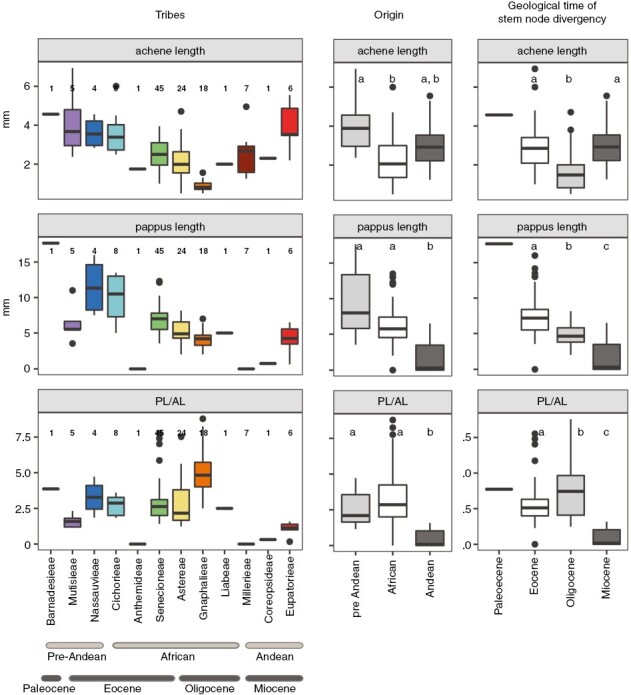
Box plots comparing diaspore trait values between tribes and within tribe groups based on origin and geological time of stem node divergency. In the tribes column, numbers at the top represent the number of species per tribe. Abbreviation: PL/AL, pappus-to-achene length ratio, as a proxy for dispersal investment. Differences between the origin and geological time groups were assessed using the Games-Howell test, and different letters indicate significant differences between the categories (*P* < 0.05), whereas the same letters indicate no significant differences.

Both achene length and dispersal investment showed strong phylogenetic signal (*P* < 0.001; Fig. 4; Supplementary Data Table S4). Among the tribes with the largest number of species were Senecioneae, Astereae and Gnaphalieae (African origin, representing 72% of all studied species), whose dispersal investment was among the highest (Fig. 5). However, high values were also found in Nassauvieae (pre-Andean origin; Fig. 5). The smallest achenes were observed in species of tribe Gnaphalieae (Fig. 5), but small achenes seemed common among most tribes with an African origin (*P* < 0.05). The largest achenes were found in Barnadesieae, Mutisieae (pre-Andean origin) and in Eupatorieae (Andean origin), and achene length was significantly smaller in tribes with stem nodes that diverged during the Oligocene (*P* < 0.05). Pappus length was significantly shorter (or non-existant) in tribes with stem nodes that were Miocene in age compared with those from the Oligocene (*P* < 0.05). Other than *Cotula mexicana*, which has a winged achene rather than a pappus, species without a pappus of bristles belong to tribes with stem nodes that diverged during the recent Miocene, i.e. Millerieae, Coreopsideae and Eupatorieae, from the Heliantheae alliance.

### Diaspore traits and distribution across the tropical Andes

**Fig. 6.**
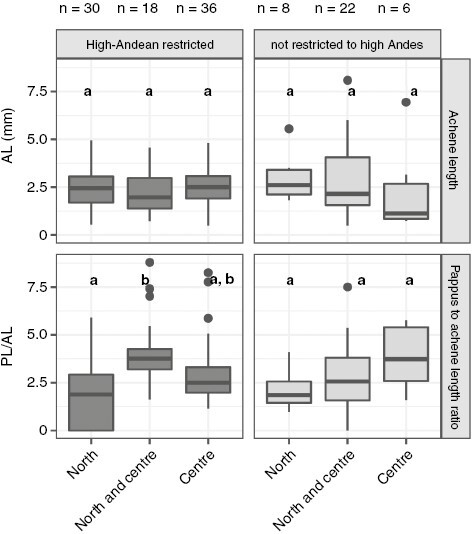
Box plots comparing seed dispersal investment [pappus-to-achene length ratio (PL/AL)] and achene length (AL) between species with distributions in different geographical Andean regions. Species can be present only in the northern Andes (north) or only in the central Andes (centre) or in both the northern and central Andes (north and centre). Results are shown separately for high-Andean-restricted species and species not restricted to the high Andes. Differences were assessed using the Games-Howell test, and different letters indicate significant differences (*P* < 0.05).

We did not find significant differences in diaspore traits between species restricted vs. non-restricted to the high Andes (Supplementary Data Fig. S8). However, within high-Andean-restricted species, those distributed across the tropical Andes (i.e. in both the northern and central Andes, *n* = 18) had a significantly higher dispersal investment (mean = 4.1) than those restricted only to the northern Andes (*n* = 30, mean = 2.1, *P* = 0.002) and marginally higher than those restricted to the central Andes (*n* = 36, mean = 2.9, *P* = 0.080) (Fig. 6). Thus, only 21% of high-Andean restricted species have crossed the Huancabamba depression. No differences were found in achene length or dispersal investment within species with broader distribution (not restricted to the high Andes) (Fig. 6), thus these species were either able or not able to cross the geographical barrier independently of their dispersal investment. Species distribution ranges did not show a strong phylogenetic signal (Supplementary Data Table S4).

## DISCUSSION

### Diversity of diaspore morphology in high-Andean Compositae

Compositae species occurring in the high tropical Andes exhibit a broad variety of diaspore morphologies, from very small achenes (the smallest being 0.5 mm) to much larger ones (8 mm). We were expecting a dominance of both small achenes and low dispersal investment (H1) because the energetic costs of developing large achenes or appendages are high, particularly in harsh environments, such as alpine habitats (Bonte *et al.*, 2012). We found support for the first part of H1, in that the majority of the studied species (75%) have small achenes, especially if we compare them with achene median lengths from other Compositae across the world (<3.3 mm). However, we also observed that 67% of our species have a pappus that is at least two times the length of the achene, suggesting high diaspore dispersal investment, which is the opposite to what we expected (second part of H1). One potential explanation is that a trade-off exists between achene size and appendage development, in which small achenes prevailed to favour longer appendages (i.e. pappus).

Species with both small achenes (<3.3 mm) and dispersal investment lower than two represent 18% of the total. These species might prioritize investment in other strategies for their populations to survive. For example, asexual reproduction (i.e. apomixis) is common among European Compositae (Körner, 2003; Pegoraro *et al.*, 2020). Although not assessed in the high-Andean Compositae, species in the region might have adopted the same strategy, which could explain the low investment in achene development. However, evidence for apomixis in some Compositae genera is sparse, and a thorough study would be required to confirm this condition more broadly (Noyes, 2007). Other strategies for surviving in the high Andes include an increased investment in leaf development, typical of giant rosettes or sclerophyllous leaves, because this confers cold resistance by avoidance of freezing and hydraulic mechanical stress (e.g. Rada *et al.*, 1985, 2019). However, only 27% of the studied species presented a rosette growth form, while the rest were mostly shrubs or herbs (data not shown).

### Abiotic and biotic factors affecting diaspore morphology in high-Andean Compositae

Our results show that both environmental conditions and evolutionary history shape diaspore morphology. We confirmed our second hypothesis (H2), which predicted that achene length and dispersal investment should be shaped by precipitation and temperature gradients. Larger achenes are found in plant communities closer to the equator. This result is also consistent with the findings of a global seed size analysis by Moles *et al.* (2007); however, in their study, highland species were poorly represented. A model simulating the effect of precipitation on seed size found that under high and constant precipitation but also under high constant aridity, large seeds should be favoured (Volis and Bohrer, 2013). Overall, precipitation increases from south to north in the tropical Andes, whereas seasonality decreases. Therefore, this gradient could explain the larger achenes found closer to the equator.

We also found elevation to be positively correlated with dispersal investment, but no correlation was found with achene length. The increase in dispersal investment at higher elevations is driven mostly by an increased pappus length rather than a decrease in achene length. Given that a better lift might be needed at higher elevations owing to lower air density (Halloy, 1989), a higher dispersal investment could be an adaptive strategy to compensate for this, rather than an actual increase in dispersal ability. Elevation gradients have been used mostly to address temperature-related effects on functional traits, but the effects of changes in air pressure or other factors, such as ultraviolet radiation, on plant diversity patterns need more attention (Halloy, 1981) at higher elevational tiers. Furthermore, studies investigating the relationship between dispersal investment and dispersal capacity are required, particularly in the context of tropical mountains. For example, this could be done by combining field observations (currently non-existent), seed weight (currently very limited to a few species) and other relevant traits, such as plant height (Thomson *et al.*, 2018). Also, further research is needed about factors not considered here that can determine diaspore morphology, such as capitulum traits and plant–animal interactions (Fu *et al.*, 2023) or wind and air humidity (e.g. Sheldon and Burrows, 1973).

A previous study, also developed in the context of the GLORIA-Andes network, found that dispersal modes are arranged along the minimum air temperature gradient in the high tropical Andes except for species dispersed by wind, which could be found all along the gradient (Tovar *et al.*, 2020). Here, we showed that within wind-dispersed species, differences in their diaspore morphology are evident along latitudinal and elevation gradients. Collection of more detailed diaspore trait data and accounting for intraspecific variation would be a fundamental next step to improve our understanding of seed ecology and community patterns across environmental gradients (Saatkamp *et al.*, 2019), particularly in the high Andes.

In addition to environmental factors and filtering, our results indicate that evolutionary history plays a role in shaping Compositae diaspore morphology. Achene length, pappus length and dispersal investment show significant phylogenetic signal, which supports our third hypothesis (H3). Our phylogenetic tree shows that 77% of species diverged in the last 10 Myr and 35% in the last 5 Myr. The lineages diverging during the Palaeocene/Eocene (before ~34 Mya) and during the Miocene (after ~23 Mya) have significantly larger achenes than those that diverged during the Oligocene (Fig. 5). This might be related to the high aridity present during these times (Herbert *et al.*, 2016; Liu *et al.*, 2020), an environmental condition that most probably selects for larger seeds (Volis and Bohrer, 2013). Among these tribes, Barnadesieae split from its sister lineage in the Cretaceous and probably originated in Patagonia in arid and warm conditions, with most species of this tribe (with a few exceptions) growing in stressed conditions (exposed to either aridity or salt stress). Likewise, arid but cold conditions prevailed when the ancestor of the tribe Millerieae diverged from its sister lineage during the Miocene. Larger achenes have been positively correlated with nutritious stores and usually produce more vigorous seedlings that are more likely to establish successfully (Chen *et al.*, 2020). However, the nutritious content of seeds could decrease owing to a thick seed coat that limits predation (Chen *et al.*, 2020), and therefore studies focusing on this for tropical alpine species are needed.

Higher dispersal investment is mostly found in lineages with African ancestors, such as Gnaphalieae, which diverged during the early Oligocene [~31 (4.4–59.3) Mya] (Mandel *et al.*, 2019). Gnaphalieae subsequently dispersed to South America, at ~15–20 Mya, and their successful colonization of open environments led to a worldwide diversification that has been attributed to their lighter achenes (Nie *et al.*, 2016). High-Andean species from Astereae and Senecioneae [stem node divergence estimate of ~31 (4.4–59.3) and ~36 (31.0–60.0) Mya, respectively], both with African origins, produce small achenes and medium pappi but, on average, dispersal investment is >2.4. Species in Gnaphalieae, Astereae and Senecioneae tribes represent 72% of the recorded Compositae species in the studied mountain summits. The high percentage of species from tribes with African ancestors found in the high Andes highlights the role of anemochory adaptations in long-distance dispersal events, across continents and in colonizing fragmented mountainous regions.

We also observed that species with novel dispersal appendages and changes in dispersal investment belong to tribes that diversified at the beginning of the Miocene, when the most important peak in diversification of Compositae occurred (Palazzesi *et al.*, 2022*a*). During this period, the tribes Millerieae, Eupatorieae and Coreopsideae diverged from their sister lineages (in the last 19 Myr) from an ancestor that re-entered the New World from Africa (Mandel *et al.*, 2019). Awns are found in the studied species belonging to Eupatorieae and Coreopsideae, which attach to animal fur and can lead to greater dispersal distances than wind-dispersed diaspores (Tamme *et al.*, 2014). Eight species (6%), mostly from tribe Millerieae, do not have a pappus and consequently have very limited dispersal ability, such as species of the genus *Espeletia* (Cuatrecasas, 2013). Generating similar phylogenomic data in other plant groups inhabiting the high Andes, such as Poaceae, and a comparison with lowland sister taxa would also provide a more comprehensive assessment of the effects of these traits on the evolution of alpine floras.

### The role of diaspore morphology in the Compositae distribution across the high Andes

We did not find support for our fourth hypothesis that suggested a low dispersal investment was favoured by species restricted to the high Andes, but we did find that very few of them crossed main barriers, such as the Huancabamba depression. First, there were no significant differences in achene length or dispersal investment between high-Andean-restricted species (*n* = 84) and those with broader distributions (*n* = 41). Second, we did not find differences in dispersal investment within species not restricted to the high Andes, and most of these species were able to cross geographical barriers independently of their diaspore dispersal investment (Fig. 6). Given that these species are also distributed in lower areas (e.g. montane forests), their suitable habitats are potentially better connected across the Andes than those restricted to the Andean grasslands.

However, we found differences in dispersal investment within the high-Andean-restricted species (Fig. 6). Only 18 of the 84 of high-Andean restricted species are present in both Páramo and Puna (i.e. northern and central Andes, respectively), but these species have significantly higher dispersal investment than 30 species restricted exclusively to the Páramo, and higher than 36 species restricted to the Puna, although this latter difference is only marginally significant. These 18 species have an average pappus length four times the length of their achene. A higher dispersal investment might have facilitated the crossing of one of the lowest points of the tropical Andes (Huancabamba depression), which divides the Páramo from the Puna (Young *et al.*, 2002). This suggests that dispersal investment is likely to contribute to shaping species distribution across the high Andes in fragmented habitats, either to facilitate (high investment) or to hinder (low investment) the crossing of geographical barriers.

Nevertheless, the fact that several Páramo endemic species have most probably speciated recently (Madriñán *et al.*, 2013) could mean that their distribution might still be expanding. To test the hypothesis that time of divergence is also potentially influencing the distribution across fragmented habitats, in addition to diaspore morphology, a complete species-level phylogenetic tree of the groups found in the high Andes would be essential in any future research investigating this topic.

## SUPPLEMENTARY DATA

Supplementary data are available at *Annals of Botany* online and consist of the following.

Figure S1: histograms showing diaspore morphological trait values for high-Andean Compositae. Figure S2: comparison of achene length data between Compositae from this study and Compositae from other studies around the world. Figure S3: phylogenomic tree reconstructed for Andean Compositae using IQ-TREE with the concatenated and partitioned nuclear dataset. Figure S4: phylogenomic tree reconstructed for Andean Compositae using ASTRAL-III for the individual gene trees reconstructed with IQ-TREE. Figure S5: phylogenomic tree reconstructed for Andean Compositae using RAxML-NG with the concatenated and partitioned nuclear dataset. Figure S6: phylogenomic tree reconstructed for Andean Compositae using ASTRAL-III for the individual gene trees reconstructed with RAxML-NG. Figure S7: phylogenomic tree reconstructed for Andean Compositae using SVDquartets approach. Figure S8: boxplots comparing trait values of species whose distribution range is restricted to the high Andes and those non-restricted, with broader distributions. Table S1: list of species used in this study. Taxonomy follows that used in the GLORIA Andes database. Table S2: list of samples used for DNA analysis. Table S3: fourth-corner analysis results. Table S4: phylogenetic signal for diaspore traits and distribution ranges. Methods S1: Supplementary Data Methods.

mcad099_suppl_Supplementary_Data

mcad099_suppl_Supplementary_Figures

mcad099_suppl_Supplementary_Tables
