# Supplementary material for: Strategies of diaspore dispersal investment in Compositae: the case of the Andean highlands
Source: Ann Bot. 2023 Jul 28;132(2):255–67. doi: 10.1093/aob/mcad099 (PMC10583198; doi:10.1093/aob/mcad099)
Supplement: mcad099_suppl_Supplementary_Data [file mcad099_suppl_supplementary_data.docx]

*Annals of Botany*

*Strategies of diaspore dispersal investment in Compositae: the case of the Andean highlands. Tovar et al. 2023*

# SUPPLEMENTARY MATERIAL EXTENDED METHODS

**Supplementary data Methods S 1 Supplementary material Methods.**

**Trait data collection for Andean Compositae**

Diaspore trait measurements (achene length, achene width and pappus length as per figure below) were collated from scientific papers and books (n = 45) as well as from herbarium specimens (n = 88). Below all the references used for trait data collection:

| Reference | Title |
| --- | --- |
| (Blake 1922) | Key to the genus *Diplostephium*, with descriptions of new species |
| (Cabrera 1939) | Las especies tucumanas del género *Senecio* (Compositae) |
| (Cabrera 1984) | Nuevas especies Bolivianas del género *Senecio* (Compositae) |
| (Calvo and Fuentes 2018) | Three new caespitose species of *Senecio* (Senecioneae, Compositae) from Central Andes |
| (Calvo *et al.* 2019) | New combinations and synonyms in discoid caespitose Andean Senecio (Senecioneae, Compositae) |
| (Calvo *et al.* 2020) | Taxonomic Revision of the Neotropical Genus *Werneria* (Compositae, Senecioneae) |
| (Calvo and Moreira-Muñoz 2020) | Taxonomic revision of *Xenophyllum* |
| (Cuatrecasas 1964) | Miscelánea sobre Flora Neotrópica |
| (Cuatrecasas 1939) | Notas a la flora de Colombia, 1 |
| (Cuatrecasas 1954) | Anuario de la Estación Altoandina de Biología Hacienda Checayani-Azángaro Puno-Perú |
| (Cuatrecasas 1967) | Estudios sobre plantas Andinas, X |
| (Davis 2010) | A systematic revision of *Chaetanthera* Ruiz & Pav., and the reinstatement of *Oriastrum* Poepp. & Endl. (Asteraceae: Mutisieae) |
| (Díaz-Piedrahita and Cuatrecasas 1999) | Asteraceas de la flora de Colombia, Senecionae - 1, Géneros *Dendrophorbium* y *Pentacalia* |
| (Dillon and Sagástegui 1991) | Flora of Peru, Family Asteraceae: Part V |
| (Dillon and Sagastegui-Alva 1996) | Revision of the Dioecious Genus *Chersodoma* Phil. (Senecioneae, Asteraceae), Including a New Species and Status Change |
| (Freire 1986) | Revisión del género *Lucilia* (Compositae, Inulae) |
| (Freire and Iharlegui 1997) | *Gamochaeta lulioana* (Asteraceae, Gnaphalieae), una Nueva Especie de los Andes de Bolivia y Perú |
| (Freire and Urtubey 2019) | *Chryselium*, a New South American Genus Segregated from *Helichrysum* (Asteraceae, Gnaphalieae)* |
| (Funk and Zermoglio 1999) | A Revision of *Chrysactinium* (Compositae: Liabeae) |
| (Hieronymus 1896) | Plantae Stuebelianae novae quas descripsit adjuvantibus aliis auetoribus (Schluss) |
| (Hieronymus 1905) | Plantae peruvianae a claro Constantino de Jelski collectae. Compositae |
| (Katinas 2012) | Revisión del género *Perezia* (Compositae) |
| (Leon H. and Gámez 2018) | Anatomía de la madera de ocho especies de *Pentacalia* (Asteraceae) en Venezuela |
| (Luebert *et al.* 2017) | Phylogeny and evolution of achenial trichomes in the *Lucilia*-group (Asteraceae) |
| (Minga *et al.* 2016) | Flora del páramo del Cajas, Ecuador |
| (Monti 2016) | Revisión taxonómica y análisis cladístico de las especies sudamericanas del género *Pseudognaphalium* Kirp. (Asteraceae, Gnaphalieae) |
| (Müller 2006) | Systematics of *Baccharis* (Compositae-Astereae) in Bolivia, including an overview of the genus |
| (Nesom 1994) | Subtribal classification of the Astereae (Asteraceae) |
| (Novara 2011) | Flora del Valle de Lerma, Asteraceae |
| (Pedraza-Peñalosa *et al.* 2004) | Chisacá, un recorrido por los Páramos Andinos |
| (Robinson 1919) | A recension of the Eupatoriums of Peru |
| (Robinson 1931) | Stevias of Ecuador |
| (Robinson 2008) | Compositae-Eupatoreae |
| (Saavedra 2018) | Efecto antiespasmódico del extracto hidroalcohólico del rizoma de *Perezia* *coerulescens* Wedd “mancharisqa” en Íleon aislado de *Cavia porcellus* “cobayo”, Ayacucho |
| (Sagastegui-Alva 1987) | A new species of *Gynoxys* (Asteraceae: Senecioneae) from northern Peru |
| (Sherff 1926) | Studies in the Genus *Bidens* |
| (Silva-Moure *et al.* 2013) | Taxonomía de *Lasiocephalus* Willd. ex Schltdl. (Asteraceae) en Venezuela |
| (Sklenář *et al.* 2005) | Flora genérica de los Páramos |
| (Standley 1915) | The Genus *Espeletia* |
| (Toro Quezada 2022) | Germinación de la *Chuquiraga* *jussieui* J.F.Gmel de los páramos del Antisana y sus implicaciones en la restauración ecológica |
| (Urtubey *et al.* 2009) | Systematics of the South American *Hypochaeris sessiliflora* Complex (Asteraceae, Cichorieae) |
| (Weddell 1855) | Expedition dans les parties centrales de l'Amerigue de Sud de Rio de Janeiro a Lima, et de Lima au Para. |
| (Zuloaga *et al.* 2014a) | Flora Argentina: Flora vascular de la República Argentina 7(1): Dicotyledoneae – Asteraceae (Anthemideae a Gnaphalieae) |
| (Zuloaga *et al.* 2014b) | Flora Argentina: Flora vascular de la República Argentina 7(3): Dicotyledoneae – Asteraceae (Senecioneae a Vernonieae) |
| (Zuloaga *et al.* 2015) | Flora Argentina: Flora vascular de la República Argentina 7(2): Dicotyledoneae – Asteraceae (Cichorieae, Helenieae a Mutisieae) |

For each species between 1 and 10 measurements were compiled from different sources (mean = 2.3 for achene and pappus length). Note that these measurements include range values where minimum and maximum values were given by the reference’s authors. In these cases, we estimated the mean value. These average value counts as 1 measurement for us as there is no information on how many achenes/pappus were used to estimate the minimum and maximum values. We got range values for achene length for 74 species and range values for pappus length for 68 species. Photos of the cypsela from specimens were taken in the following herbaria: Herbario Nacional de La Paz (LPB), Herbario de la Universidad Nacional de Cajamarca (CPUN), Jardín Botánico de Bogotá (JBB), Herbario Nacional Colombiano (COL) del Instituto de Ciencias Naturales (Universidad Nacional de Colombia), Herbario Universidad Mayor de San Marcos (USM), Herbario de la Pontificia Universidad Católica del Ecuador (PUCE), Herbario de la Molina (MOL), Herbarium Naturalis Biodiversity Centre (U), Herbarium Smithsonian Institution (US) and Herbarium Aarhus University (AAU). Using the software ImageJ (Image J version 1.50i, NIH, USA), the longest length and width dimensions of the achene and pappus were recorded. For species in which infertile achenes were present (i.e., male or functionally staminate), measurements were only recorded from those that were identified as fertile (pistillate or hermaphrodite). Only measurements of developed achenes were recorded, except for 5 species for which only achenes with flowers were found: *Senecio anconquijae*, *Senecio humillimus*, *Senecio neeanus*, *Senecio rufescens*, *Senecio spinosus*. Scales were not observed on the images of some species (n = 4), in which case the ratio of pappus vs achene length was recorded.


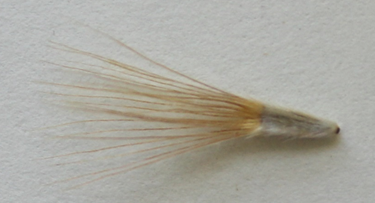


PL

AL

AW

**Achene length data collection from other Compositae around the world**

We collected information for achene length for Compositae species from three different sources: the TRY database (Kattge *et al.* 2020), the Seed Information Database (Royal Botanic Gardens, Kew 2023) and a paper from the alpine ecosystems of New Zealand (Richardson *et al.* 2012). After checking the records to clean erroneous records, we kept the data for 155 Compositae species distributed across the world and in different ecosystems (see table below). When a species had more than one record, we estimated the mean value; when a range value was provided, we took the mid-point value.

| Source | Reference | # species | Geographic area |
| --- | --- | --- | --- |
| Richardson et al. 2012 | (Richardson *et al.* 2012) | 22 | Alpine region of New Zealand |
| SID | (Royal Botanic Gardens, Kew 2023) | 23 | 70% from south western Africa, rest, around the world (North America, Mediterranean, Australia) |
| TRY | (Fitter and Peat 1994) | 104 | UK flora |
| TRY | (Kühn *et al.* 2004) | 1 | German flora |
| TRY | (Schroeder-Georgi *et al.* 2016) | 10 | Central European mesic grasslands |
| TRY | Unpublished | 1 | Europe |

**References**

**Blake SF**. **1922**. Key to the genus *Diplostephium*, with descriptions of new species. *Contributions from the United States National Herbarium* **24**: 65–86.

**Cabrera AL**. **1939**. Las especies tucumanas del género *Senecio* (Compositae). *Lilloa* **5**: 65–120.

**Cabrera AL**. **1984**. Nuevas especies Bolivianas del género *Senecio* (Compositae). *Hickenia boletin del Darwinion* **2**: 13–25.

**Calvo J, Fuentes AF**. **2018**. Three new caespitose species of *Senecio* (Senecioneae, Compositae) from Central Andes. *Phytotaxa* **375**: 70.

**Calvo J, Granda A, Funk VA**. **2019**. New combinations and synonyms in discoid caespitose Andean *Senecio* (Senecioneae, Compositae). *PhytoKeys* **132**: 111–130.

**Calvo J, Moreira-Muñoz A**. **2020**. Taxonomic revision of the Andean genus *Xenophyllum* (Compositae, Senecioneae). *PhytoKeys* **158**: 1–106.

**Calvo J, Moreira-Muñoz A, Funk VA**. **2020**. *Taxonomic revision of the Neotropical genus* Werneria *(Compositae, Senecioneae)*. Smithsonian Contributions to Botany Number III. Washington D.C.: Smithsonian Scholarly Press.

**Cuatrecasas J**. **1939**. Notas a la flora de Colombia I. : 247–250.

**Cuatrecasas J**. **1954**. *Mniodes pulvinata* Cuatrec. *Anuario de la Estación Altoandina de Biología Hacienda Checayani-Azángaro Puno-Perú* **1**.

**Cuatrecasas J**. **1964**. Miscelánea sobre Flora Neotrópica. *Ciencia Revista Hispanoamericana de Ciencias Puras y Aplicadas* **23**: 137–151.

**Cuatrecasas J**. **1967**. Estudio sobre plantas andinas. *Caldasia* **10**: 3–26.

**Davies AMR**. **2010**. A systematic revision of *Chaetanthera* Ruiz & Pav., and the reinstatement of *Oriastrum* Poepp. & Endl. (Asteraceae: Mutisieae). PhD Thesis, University of Munich, Germany

**Díaz-Piedrahita S, Cuatrecasas J**. **1999**. *Asteraceas de la flora de Colombia, Senecionae – 1, Géneros* Dendrophorbium *y* Pentacalia. Bogota, Colombia: Academia Colombiana de Ciencias Exactas, Físicas y Naturales.

**Dillon MO, Sagástegui A**. **1991**. *Flora of Peru. Family Asteraceae, Part V* (JF MacBride, Ed.). Chicago, USA: Field Museum of Natural History.

**Dillon MO, Sagastegui-Alva A**. **1996**. Revision of the dioecious genus *Chersodoma* Phil. (Senecioneae, Asteraceae), including a new species and status change. *Brittonia* **48**: 582.

**Fitter AH, Peat HJ**. **1994**. The ecological Flora database. *The Journal of Ecology* **82**: 415.

**Freire SE**. **1986**. Revisión del género *Lucilia* (Compositae, Inulae). *Darwiniana, Nueva serie* **27**: 431–490.

**Freire SE, Iharlegui L**. **1997**. *Gamochaeta lulioana* (Asteraceae, Gnaphalieae), una nueva especie de los Andes de Bolivia y Perú. *Novon* **7**: 32.

**Freire SE, Urtubey E**. **2019**. *Chryselium*, a new South American genus segregated from *Helichrysum* (Asteraceae, Gnaphalieae). *Systematic Botany* **44**: 233–242.

**Funk VA, Zermoglio MF**. **1999**. A revision of *Chrysactinium* (Compositae: Liabeae). *Systematic Botany* **24**: 323.

**Hieronymus G**. **1896**. Plantae Stuebelianae novae quas descripsit adjuvantibus aliis auetoribus (Schluss). *Botanische Jahrbücher fur Systematik, Pflanzengeschichte und Pflanzengeographie* **21**: 369–378.

**Hieronymus G**. **1905**. Plantae peruvianae a claro Constantino de Jelski collectae. Compositae. *Botanische Jahrbücher fur Systematik, Pflanzengeschichte und Pflanzengeographie* **36**: 455–513.

**Katinas L**. **2012**. Sistemática de plantas vasculares. Reivisión del género *Perezia* (Compositae). *Bol. Soc. Argent. Bot.* **47**: 159–261.

**Kattge J, Bönisch G, Díaz S, *et al.*** **2020**. TRY plant trait database – enhanced coverage and open access. *Global Ecology and Biogeography* **26**: 119–188.

**Kühn I, Durka W, Klotz S**. **2004**. BiolFlor – a new plant-trait database as a tool for plant invasion ecology: BiolFlor – a plant-trait database. *Diversity and Distributions* **10**: 363–365.

**Leon H. WJ, Gámez LE**. **2018**. Anatomía de la madera de ocho especies de *Pentacalia* (Asteraceae) en Venezuela. *Caldasia* **40**: 41–53.

**Luebert F, Moreira-Muñoz A, Wilke K, Dillon MO**. **2017**. Phylogeny and evolution of achenial trichomes in the *Lucilia*‐group (Asteraceae: Gnaphalieae) and their systematic significance. *TAXON* **66**: 1184–1199.

**Minga D, Ansaloni R, Verdugo A, Ulloa Ulloa C**. **2016**. *Flora del páramo de Cajas, Ecuador*. Cuenca, Ecuador: Universidad del Azuay.

**Monti C**. **2016**. Revisión taxonómica y análisis cladístico de las especies sudamericanas del género *Pseudognaphalium* Kirp. (Asteraceae, Gnaphalieae). PhD Thesis, Universidad Nacional de la Plata, Argentina.

**Müller J**. **2006**. *Systematics of* Baccharis *(Compositae-Astereae) in Bolivia, including an overview of the genus*. American Society of Plant Taxonomists.

**Nesom G**. **1994**. Subtribal classification of the Astereae (Asteraceae). *Phytologia* **196**: 193–274.

**Novara LJ**. **2011**. Flora del Valle de Lerma: Asteraceae Bercht. & J. Presl. Tribu VIII. Senecioneae Cass. *Aportes botánicos de Salta. Serie Flora* **11**: 1–75.

**Pedraza-Peñalosa P, Betancur J, Franco-Rosselli P**. **2004**. *Chisacá, un recorrido por los páramos andinos*. Bogota, Colombia: Universidad de Colombia and Instituto Himboldt.

**Richardson SJ, Williams PA, Mason NWH, *et al.*** **2012**. Rare species drive local trait diversity in two geographically disjunct examples of a naturally rare alpine ecosystem in New Zealand (F de Bello, Ed.). *Journal of Vegetation Science* **23**: 626–639.

**Robinson BL**. **1919**. A recension of the Eupatoriums of Peru. *Contributions from the Gray Herbarium of Harvard University* **60**: 42–88.

**Robinson BL**. **1931**. Stevias of Ecuador. *Contributions from the Gray Herbarium of Harvard University* **96**: 28–36.

**Robinson H**. **2008**. *Compositae-Eupatorieae*. In: Flora of Ecuador 83 part 190(3). Botanical Institute, Goteborg University, 1–349.

**Royal Botanic Gardens, Kew**. **2023**. *Seed Information Database*. <https://data.kew.org/sid/sidsearch.html>.

**Saavedra CR**. **2018**. Efecto antiespasmódico del extracto hidroalcohólico del rizoma de *Perezia coerulescens* Wedd “mancharisqa” en Íleon aislado de Cavia porcellus “cobayo”, Ayacucho. Undergraduate Thesis, Universidad Nacional San Cristóbal de Huamanga, Peru.

**Sagastegui-Alva A**. **1987**. A New Species of *Gynoxys* (Asteraceae: Senecioneae) from Northern Peru. *Brittonia* **39**: 432–435.

**Schroeder-Georgi T, Wirth C, Nadrowski K, Meyer ST, Mommer L, Weigelt A**. **2016**. From pots to plots: hierarchical trait-based prediction of plant performance in a mesic grassland (D Gibson, Ed.). *Journal of Ecology* **104**: 206–218.

**Sherff EE**. **1926**. Studies in the genus *Bidens*. VII. *Botanical Gazette* **81**: 25–54.

**Silva-Moure K, Torrecilla P, Lapp M**. **2013**. Taxonomía de *Lasiocephalus* Willd. ex Schltdl. (Asteraceae) en Venezuela. *Ernstia* **23**: 91–118.

**Sklenář P, Luteyn JL, Ulloa C, Jorgensen PM, Dillon MO**. **2005**. *Flora genérica de los Páramos. Guía ilustrada de las plantas vasculares*. New York: The New York Botanical Garden.

**Standley PC**. **1915**. The genus *Espeletia*. *American Journal of Botany* **2**: 468–486.

**Toro Quezada CA**. **2022**. Germinación de la Chuquiragua *Chuqiraga jussieui* J. F. Gmel de los páramos del Antisana y sus implicaciones en la restauración ecológica. Undergraduate Thesis, Universidad del Azuay, Ecuador.

**Urtubey E, Stuessy TF, Tremetsberger K**. **2009**. Systematics of the South American *Hypochaeris sessiliflora* complex (Asteraceae, Cichorieae). *Annals of the Missouri Botanical Garden* **96**: 685–714.

**Weddell HA**. **1855**. *Expedition dans les parties centrales de l’Amerigue de Sud de Rio de Janeiro a Lima, et de Lima au Para. Chloris andina. Essai d’une flore de la région alpine des Cordillères de l’Amérique du Sud*. Paris, France.

**Zuloaga FO, Belgrano MJ, Anton AM (Eds.)**. **2014a**. *Flora Argentina: Flora vascular de la República Argentina 7(1): Dicotyledoneae-Asteraceae (Anthemideae a Gnaphalieae)*. Instituto de Botánica Darwinion.

**Zuloaga FO, Belgrano MJ, Anton AM (Eds.)**. **2014b**. *Flora Argentina: Flora vascular de la República Argentina 7(3): Dicotyledoneae-Asteraceae (Senecioneae a Vernonieae)*. Instituto de Botánica Darwinion.

**Zuloaga FO, Belgrano MJ, Anton AM (Eds.)**. **2015**. *Flora Argentina: Flora vascular de la República Argentina 7(2): Dicotyledoneae-Asteraceae (Cichorieae, Helenieae aMutisieae)*. Instituto de Botánica Darwinion.
