# Supplementary material for: Strategies of diaspore dispersal investment in Compositae: the case of the Andean highlands
Source: Ann Bot. 2023 Jul 28;132(2):255–67. doi: 10.1093/aob/mcad099 (PMC10583198; doi:10.1093/aob/mcad099)

*Annals of Botany*

*Strategies of diaspore dispersal investment in Compositae: the case of the Andean highlands. Tovar et al. 2023*

# SUPPLEMENTARY MATERIAL FIGURES

Supplementary data Fig. S1 Histograms showing diaspore morphological trait values for high-Andean Compositae. A total of 122 species were used in the achene length histogram, 104 for achene width, 125 for pappus length, and 123 for dispersal investment measured as the pappus-to-achene length ratio (PL/AL).


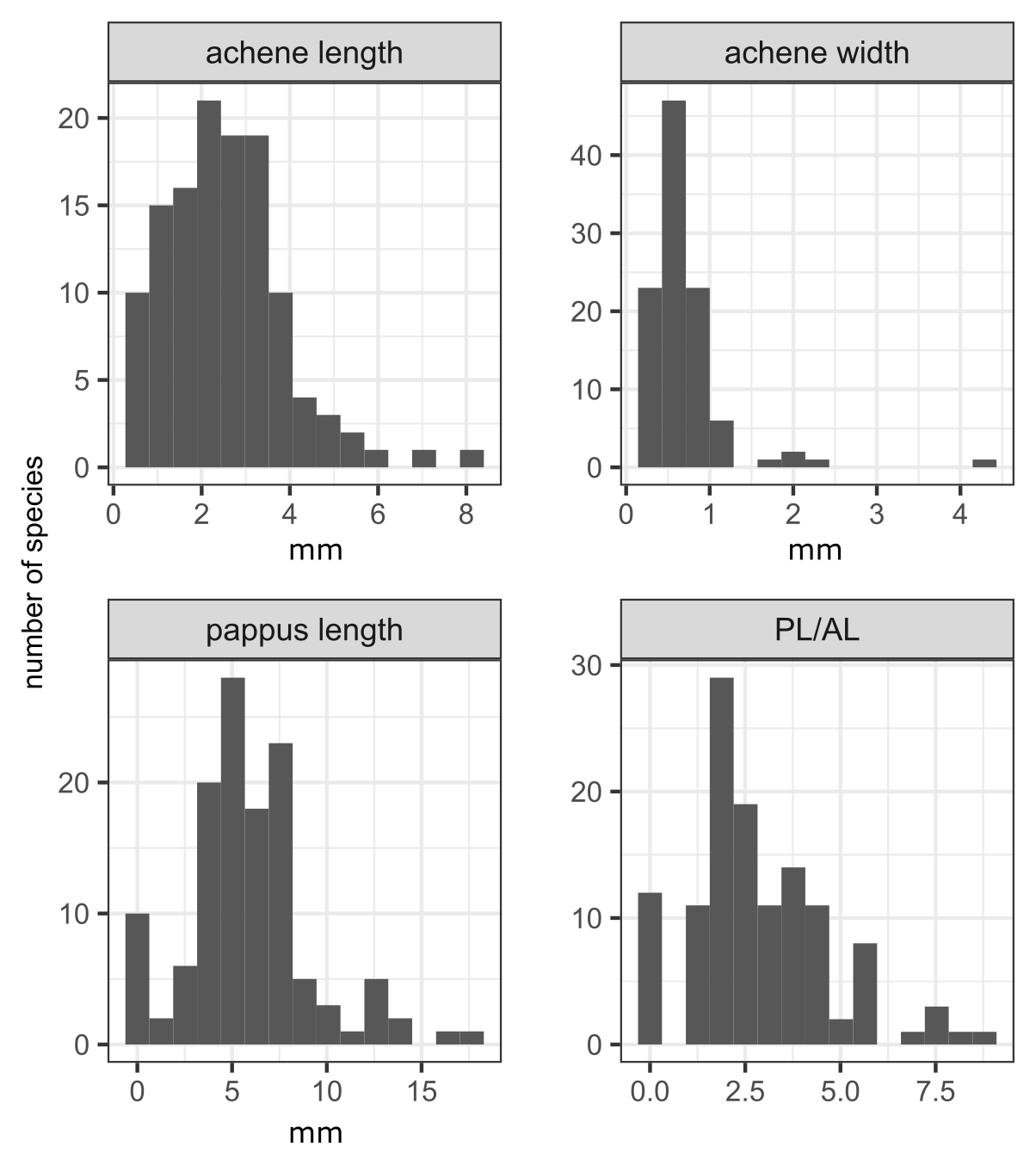


Supplementary data Fig. S2 Comparison of achene length data between Compositae from this study (Compositae GLORIA, n = 122) and Compositae from other studies around the world (other Compositae, n = 155). a) Histograms of achene length for each dataset showing the median value (Other Compositae = 3.3 mm, Compositae GLORIA = 2.4 mm). b) Boxplot comparing achene length between the two datasets. Wilcoxon test was significant (*** indicates p-value < 0.001).


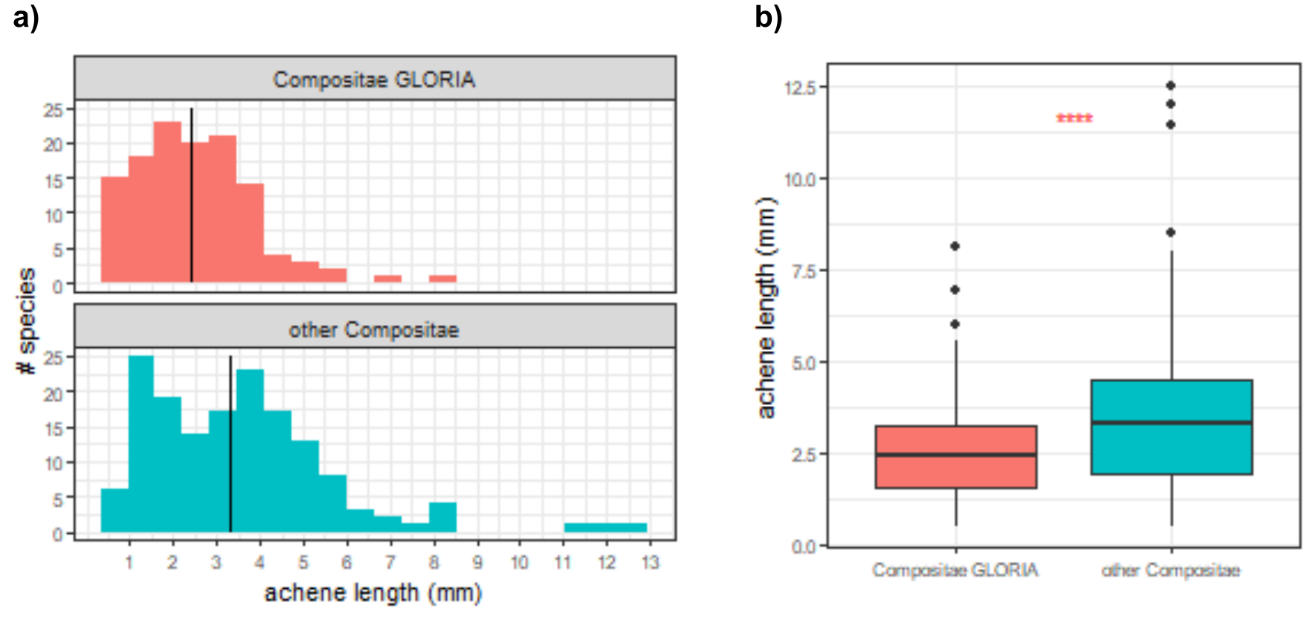


Supplementary data Fig. S3 Phylogenomic tree reconstructed for Andean Compositae using IQTREE with the concatenated and partitioned nuclear dataset, with their respective bootstrap support values shown at the nodes.


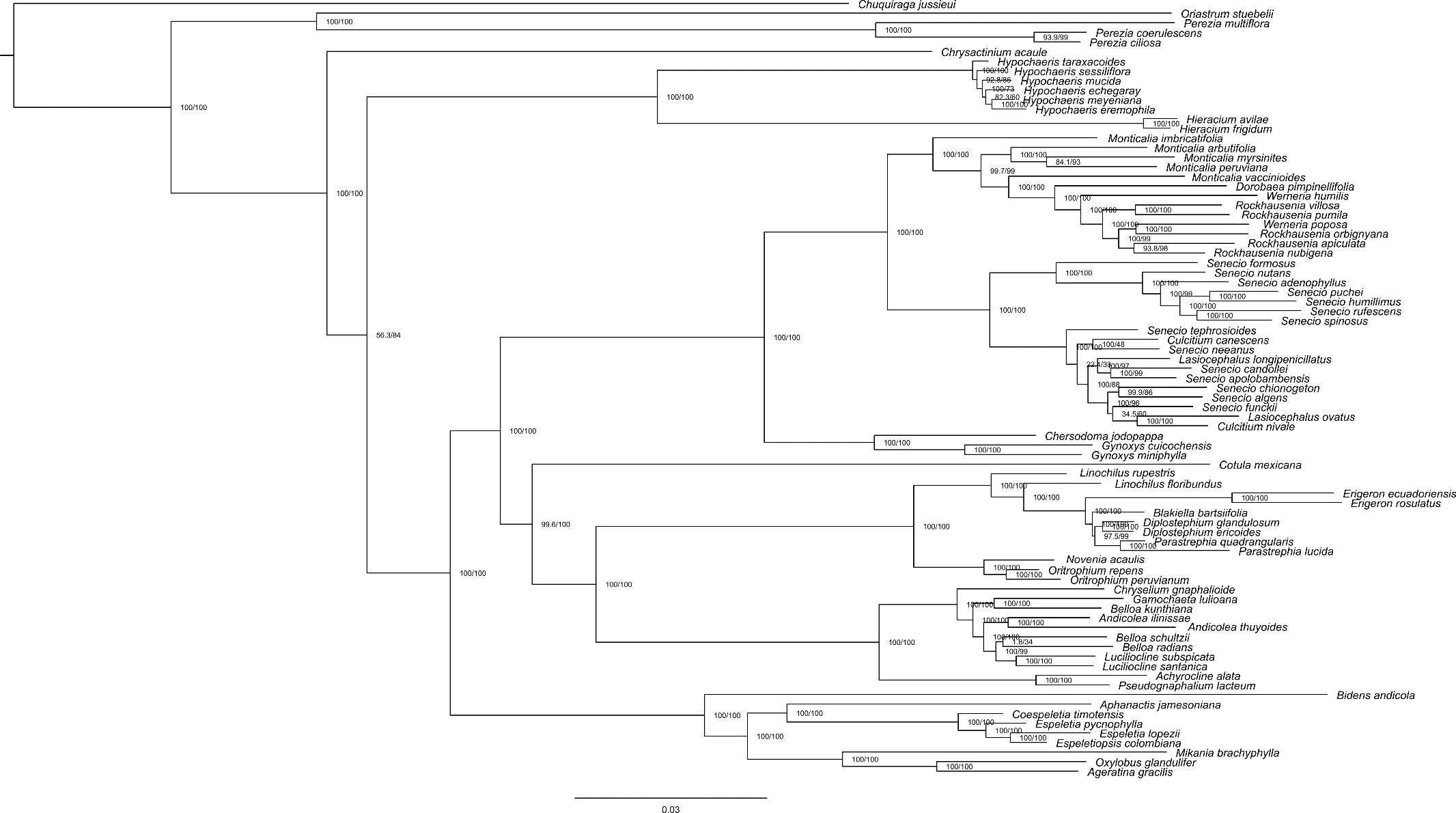


Supplementary data Fig. S4 Phylogenomic tree reconstructed for Andean Compositae using ASTRAL-III for the individual gene trees reconstructed with IQTREE, showing local posterior probability values at nodes.


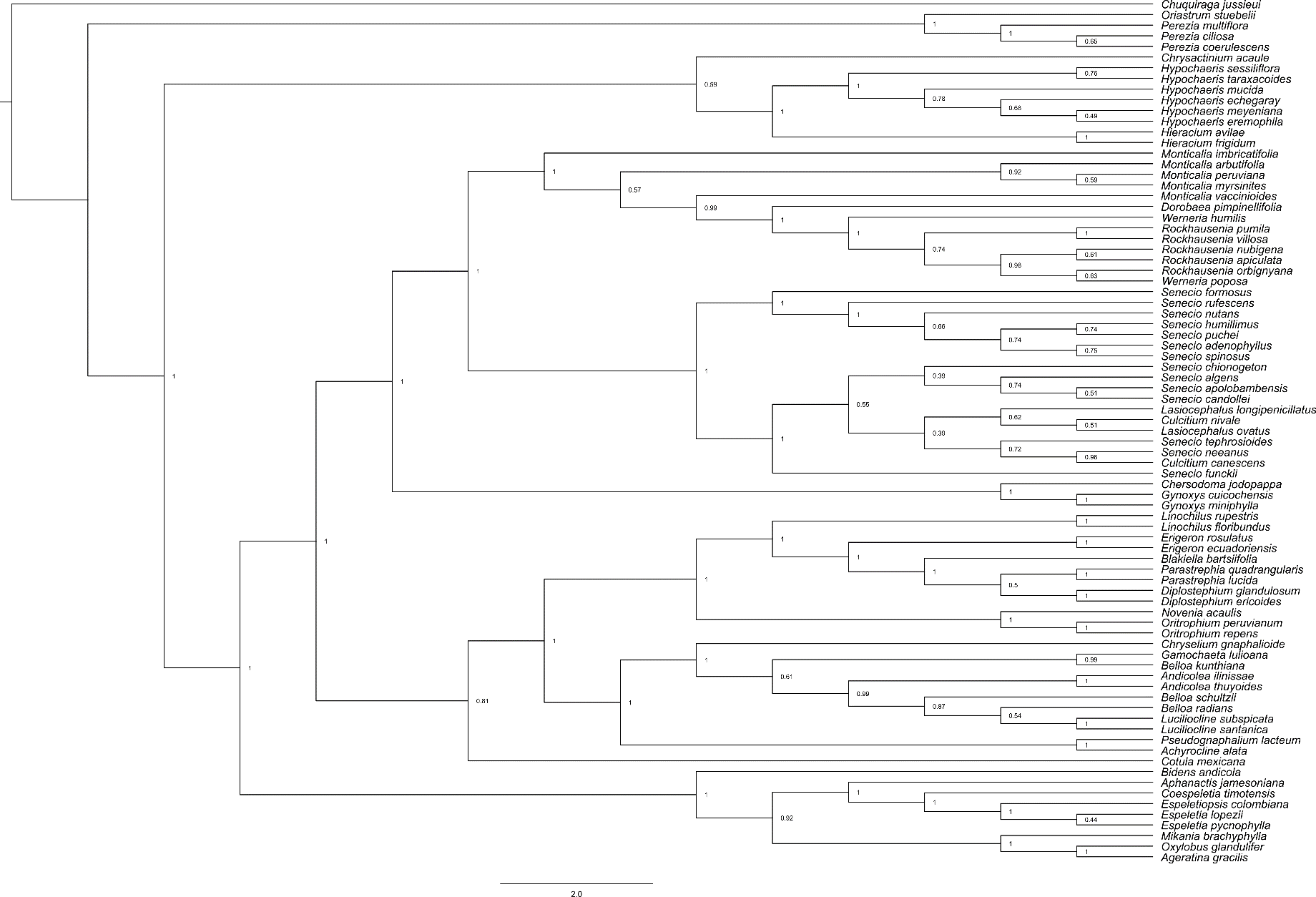


Supplementary data Fig. S5 Phylogenomic tree reconstructed for Andean Compositae using RAxML-ng with the concatenated and partitioned nuclear dataset, with bootstrap support values shown at nodes.


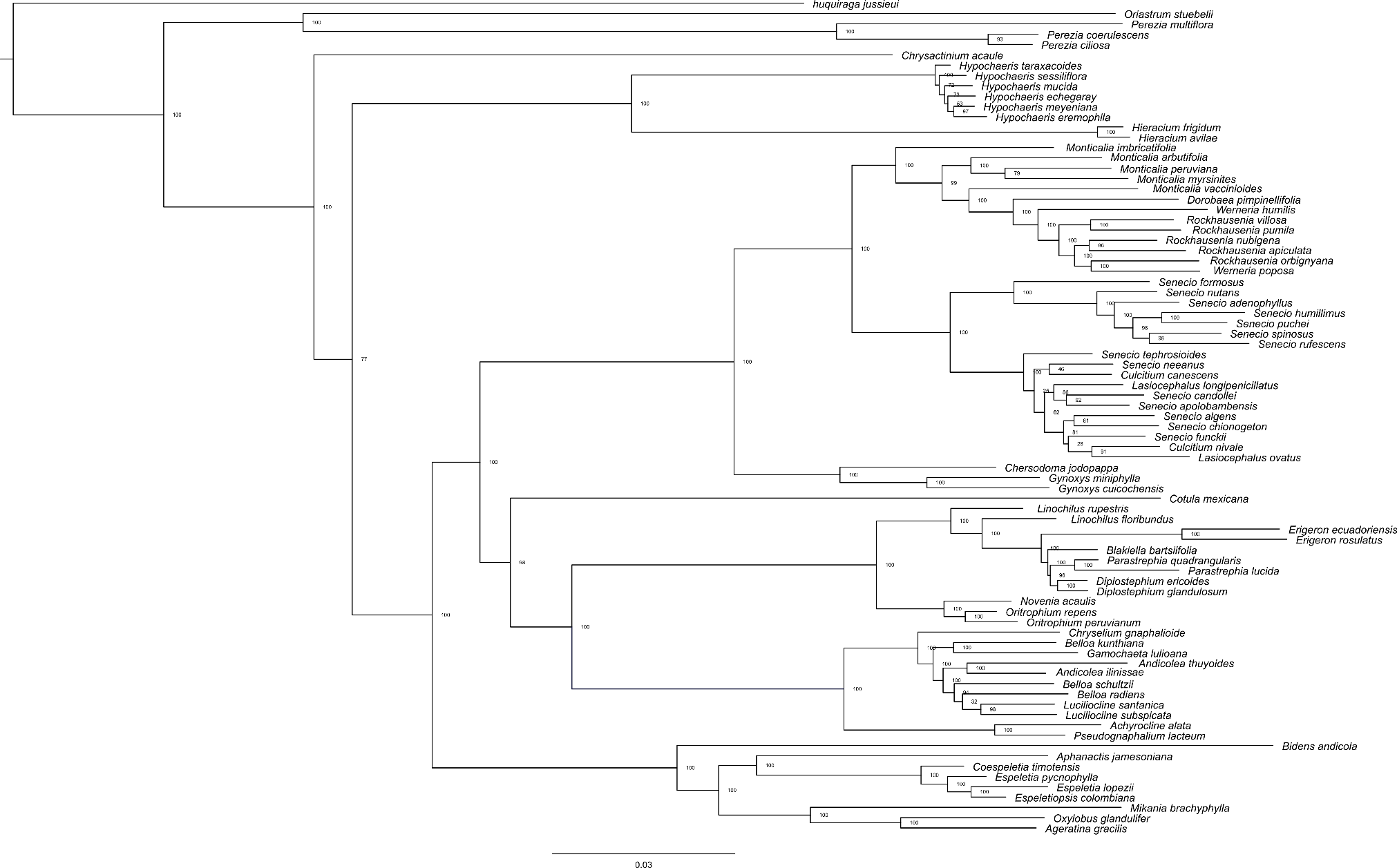


Supplementary data Fig. S6 Phylogenomic tree reconstructed for Andean Compositae using ASTRAL-III for the individual gene trees reconstructed with RAxML-ng, showing local posterior probability values shown at the nodes.


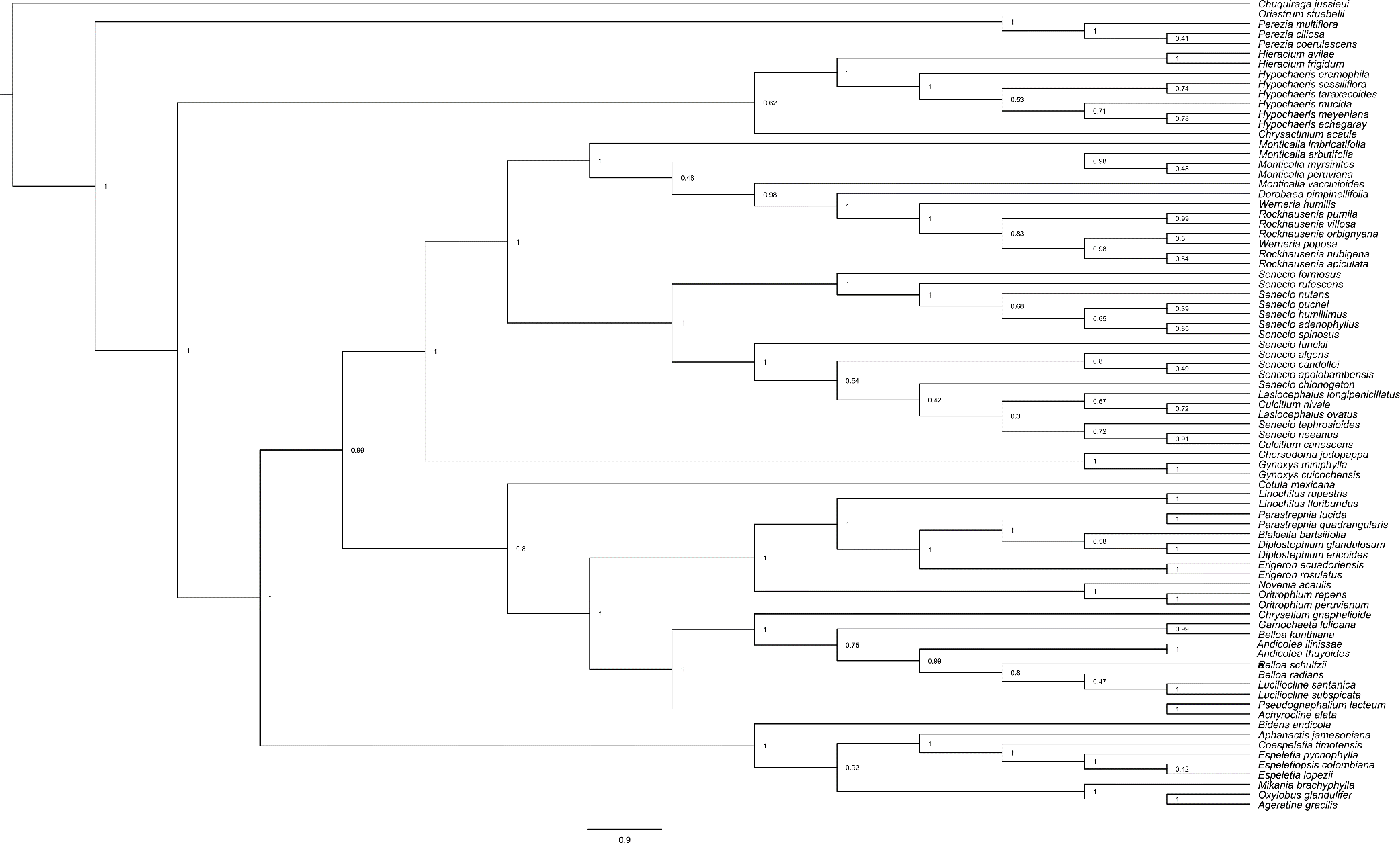


Supplementary data Fig. S7 Phylogenomic tree reconstructed for Andean Compositae using SVDQUARTETS approach showing bootstrap values at nodes.


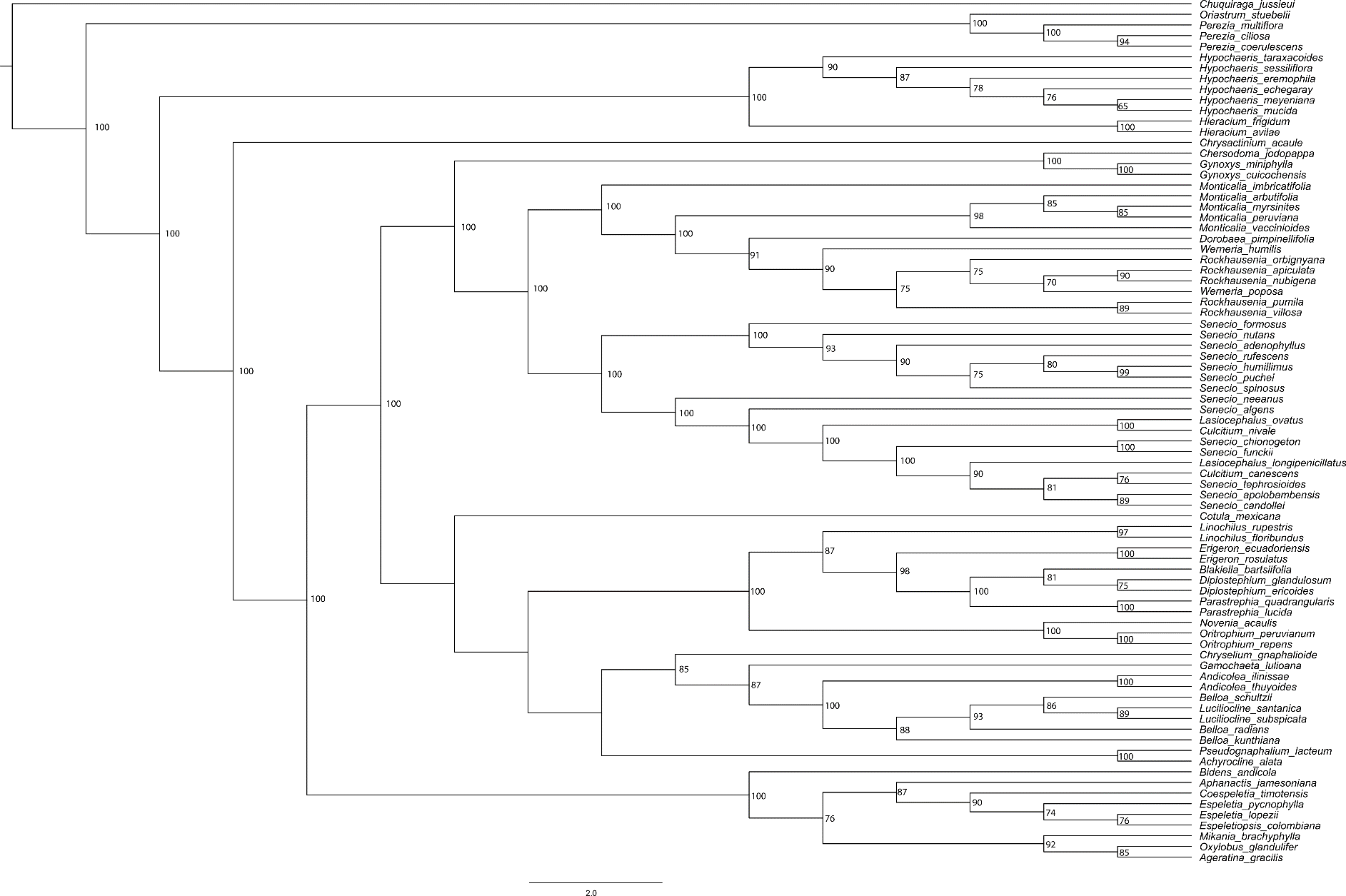


Supplementary data Fig. S8 Boxplots comparing trait values of species whose distribution range is restricted to the high Andes (n=84) and those non-restricted with broader distributions (n=41). No significant differences were found using a Wilcoxon rank sum test. PL/AL = pappus-to-achene length ratio used as a proxy for dispersal investment.


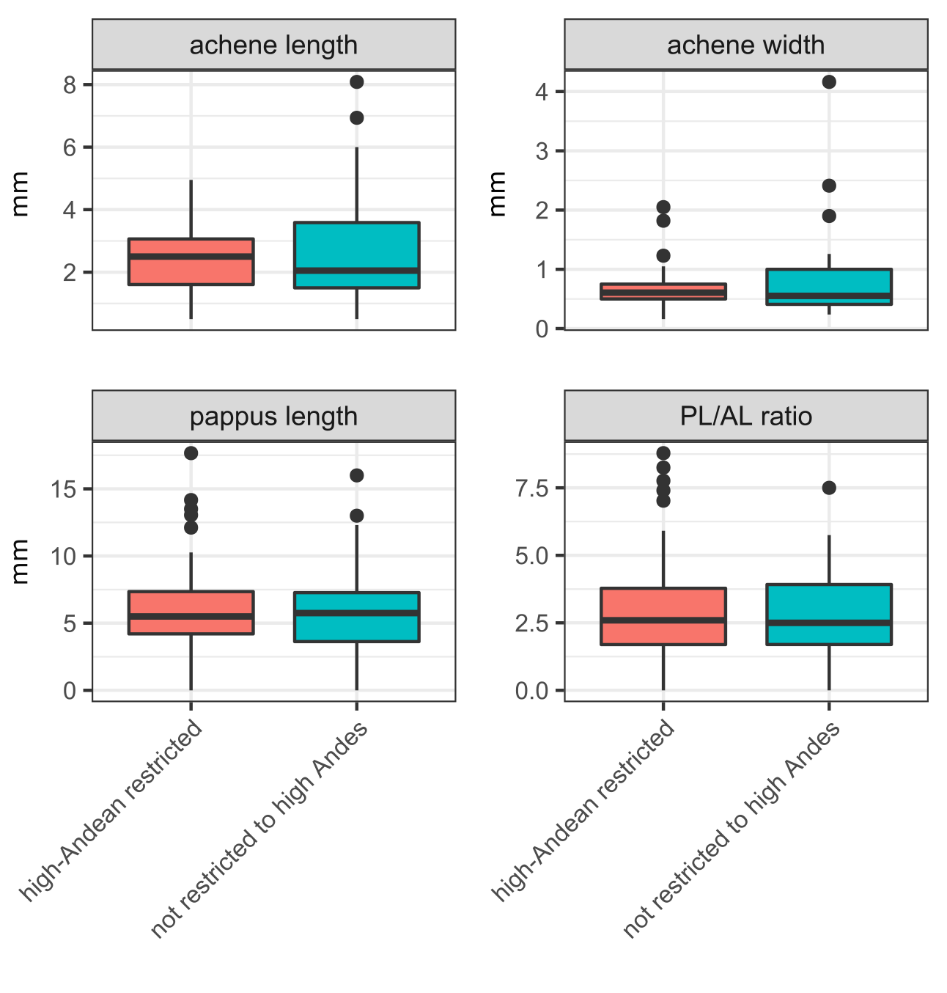

Supplement: mcad099_suppl_Supplementary_Figures [file mcad099_suppl_supplementary_figures.docx]
