# Supplementary material for: Strategies of diaspore dispersal investment in Compositae: the case of the Andean highlands
Source: Ann Bot. 2023 Jul 28;132(2):255–67. doi: 10.1093/aob/mcad099 (PMC10583198; doi:10.1093/aob/mcad099)
Supplement: mcad099_suppl_Supplementary_Tables [file mcad099_suppl_supplementary_tables.docx]

*Annals of Botany*

*Strategies of diaspore dispersal investment in Compositae: the case of the Andean highlands. Tovar et al. 2023*

# SUPPLEMENTARY MATERIAL TABLES

Supplementary data Table S1 List of species used in this study. Taxonomy follows that used in the GLORIA Andes database. Trait values represent mean values of all measurements found for a given species (see Supplementary Materials S1). AL = achene length, AW = achene width, PL = pappus length, PL/AL = pappus-to-achene length ratio. Values in brackets indicate the number of measurements used to estimate the mean. * indicates that at least one of these measurement is a mean value of a range given in a reference, which indicates more than one diaspore was measured to estimate this range. Values of PL/AL shown in the table were used only when AL and PL were not available to estimate the ratio. Names have been updated from previous publications according to the latest nomenclatural valid names. Names in brackets are the names that were used in previous GLORIA Andes papers (now synonyms).

| Tribe | Taxon name | Taxon authority | Pappus type | Mean AL (mm) | Mean AW (mm) | Mean PL (mm) | Mean PL/AL |
| --- | --- | --- | --- | --- | --- | --- | --- |
| Anthemideae | *Cotula mexicana* | (DC.) Cabrera | winged achene | 1.75 (2)* | 1.9 (1) | 0 (1) |  |
| Astereae | *Baccharis alpina* | Kunth | bristle | 1.48 (3)* | 0.49 (2)* | 5.4 (1)* |  |
| Astereae | *Baccharis arbutifolia* | Vahl | bristle |  |  | 3.85 (1)* |  |
| Astereae | *Baccharis caespitosa* | Pers. | bristle | 1.75 (1)* |  | 5.67 (2) |  |
| Astereae | *Baccharis genistelloides* | (Lam.) Pers. | bristle | 1.67 (2)* | 0.41 (2)* | 5.85 (2) |  |
| Astereae | *Baccharis nitida* | Pers. | bristle | 1.53 (3)* |  | 7 (2) |  |
| Astereae | *Baccharis prunifolia* | Kunth | bristle | 0.5 (1) |  | 3.75 (1)* |  |
| Astereae | *Baccharis tola* | Phil. | bristle | 2.05 (2)* | 0.7 (1)* | 8.15 (1)* |  |
| Astereae | *Baccharis tricuneata* (L.f.) var. *procumbens* | Cuatrec. | bristle | 0.94 (3)* | 0.31 (2) | 4.33 (4) |  |
| Astereae | *Blakiella bartsiifolia* | (S.F.Blake) Cuatrec. | bristle | 3.8 (1)* | 0.55 (1)* | 4.7 (1) |  |
| Astereae | *Conyza deserticola* | Phil. | bristle | 1.56 (2)* | 0.43 (2) | 3.51 (2)* |  |
| Astereae | *Diplostephium empetrifolium* | S.F.Blake | bristle | 1.83 (2)* |  | 6.5 (2)* |  |
| Astereae | *Diplostephium ericoides* | (Lam.) Cabrera | bristle | 0.84 (1) | 0.29 (1) | 4.73 (1) |  |
| Astereae | *Diplostephium glandulosum* | Hieron. | bristle | 1.91 (7) | 0.37 (7) | 7.81 (7) |  |
| Astereae | *Diplostephium macrocephalum* | S.F.Blake | bristle | 2 (1) |  | 7.5 (1)* |  |
| Astereae | *Erigeron ecuadoriensis* | Hieron. | bristle | 4.71 (3) | 0.8 (3) | 6.75 (3) |  |
| Astereae | *Erigeron rosulatus* | Wedd. | bristle | 2 (1) | 0.4 (1) | 3.38 (2)* |  |
| Astereae | *Hinterhubera columbica* | Sch.Bip. | bristle | 2.6 (1) | 0.7 (1) | 4.15 (1) |  |
| Astereae | *Linochilus floribundus*  *(Diplostephium floribundum)* | Benth | bristle | 2.41 (7) | 0.6 (7) | 4.53 (7) |  |
| Astereae | *Linochilus rupestris*  *(Diplostephium rupestre)* | (Kunth) Saldivia & O.M.Vargas | bristle | 3.52 (5)* | 1 (4) | 7.31 (6) |  |
| Astereae | *Hysterionica pulchella* | Cabrera | bristle | 1 (1) | 0.5 (1) | 2 (1) |  |
| Astereae | *Novenia acaulis* | (Wedd. ex Benth. & Hook. f.) S.E.Freire & F. Hellwig | bristle | 3.08 (2)* | 1 (1)* | 4.72 (2)* |  |
| Astereae | *Oritrophium peruvianum* | (Lam.) Cuatrec. | bristle | 3.74 (3)* | 0.58 (2)* | 6.05 (3)* |  |
| Astereae | *Oritrophium repens* | (Kunth) Cuatrec. | bristle | 1.98 (2)* | 0.49 (2)* | 2.95 (2) |  |
| Astereae | *Parastrephia lucida* | (Meyen) Cabrera | bristle | 2.75 (1)* |  | 4.63 (2)* |  |
| Astereae | *Parastrephia quadrangularis* | (Meyen) Cabrera | bristle | 2.5 (1)* |  | 5.03 (2)* |  |
| Astereae | *Plagiocheilus peduncularis* | (Kunth) Wedd. | epappose |  |  | 0 (1) | 0 (1) |
| Barnadesieae | *Chuquiraga jussieui* | J.F.Gmel. | bristle | 4.57 (3) | 1.82 (3) | 17.66 (2) |  |
| Cichorieae | *Hieracium avilae* | Kunth | bristle | 2.8 (3) | 0.36 (2) | 5.14 (3)* |  |
| Cichorieae | *Hieracium frigidum* | Wedd. | bristle | 2.49 (5)* | 0.52 (4) | 5 (5)* |  |
| Cichorieae | *Hypochaeris echegarayi* | Hieron. | bristle | 4.5 (1)* | 1.15 (1) | 13 (1)* |  |
| Cichorieae | *Hypochaeris eremophila* | Cabrera | bristle | 3.15 (1)* | 0.77 (1) | 9 (1)* |  |
| Cichorieae | *Hypochaeris meyeniana* | (Walp.) Griseb. | bristle | 6 (1)* | 1.26 (1) | 12 (1)* |  |
| Cichorieae | *Hypochaeris mucida* | Domke | bristle | 2.5 (1)* | 0.66 (1) | 8 (1) |  |
| Cichorieae | *Hypochaeris sessiliflora* | Kunth | bristle | 3.87 (7)* | 0.42 (6) | 13.5 (7)* |  |
| Cichorieae | *Hypochaeris taraxacoides* | (Meyen & Walp.) Ball | bristle | 3.62 (2)* | 0.51 (2) | 13.05 (2)* |  |
| Coreopsideae | *Bidens andicola* | Kunth | awns | 8.09 (4)* | 0.56 (5)* | 1.24 (3)* |  |
| Coreopsideae | *Coreopsis venusta* | Kunth | awns | 2.3 (1) | 0.45 (1) | 0.72 (1) |  |
| Eupatorieae | *Ageratina cutervensis* | (Hieron.) R.M.King & H.Rob. | bristle | 3.5 (1) |  | 3.45 (1) |  |
| Eupatorieae | *Ageratina gracilis* | (Kunth) R.M.King & H.Rob. | bristle | 2.2 (5)* | 0.33 (4) | 3.48 (6)* |  |
| Eupatorieae | *Mikania brachyphylla* | Hieron. | bristle | 5.55 (2) | 1.25 (2) | 5.75 (4) |  |
| Eupatorieae | *Mikania featherstonei* | B.L.Rob. | bristle | 5.3 (1) | 0.8 (1) | 6.5 (1) |  |
| Eupatorieae | *Oxylobus glandulifer* | (Sch.Bip. ex Hemsl.) A.Gray ex Klatt | scale | 3.55 (1)* | 0.55 (1)* | 0.6 (1) |  |
| Eupatorieae | *Stevia andina* | B.L.Rob. | awns | 3.47 (3) | 0.6 (2) | 5 (1) |  |
| Gnaphalieae | *Achyrocline alata* | DC. | bristle | 0.69 (2)* | 0.27 (1) | 2.73 (2) | |
| Gnaphalieae | *Andicolea ilinissae*  *(Loricaria ilinissae)* | (Benth.) Mayta & Molinari | bristle | 1.56 (2) | 0.65 (2) | 4.36 (4) |  |
| Gnaphalieae | *Andicolea thuyoides*  *(Loricaria thuyoides)* | (Lam.) Mayta & Molinari | bristle | 1.03 (3)* | 0.48 (2) | 4.37 (5) |  |
| Gnaphalieae | *Belloa kunthiana* | (DC.) Anderb. & S.E.Freire | bristle | 0.73 (7)* | 0.22 (5) | 6.43 (2)* |  |
| Gnaphalieae | *Belloa pickeringii* | (A.Gray) Sagást. & M.O.Dillon | bristle | 0.5 (1)* |  | 4.13 (2)* |  |
| Gnaphalieae | *Belloa piptolepis* | (Wedd.) Cabrera | bristle | 0.82 (4)* | 0.3 (2) | 4.5 (2)* |  |
| Gnaphalieae | *Belloa radians* | (Benth.) Sagást. & M.O.Dillon | bristle | 0.82 (3)* | 0.3 (2)* | 4.76 (6)* |  |
| Gnaphalieae | *Belloa schultzii* | (Wedd.) Cabrera | bristle | 0.88 (2)* | 0.35 (2)* | 3.85 (2)* |  |
| Gnaphalieae | *Chryselium gnaphalioides* | (Kunth) Urtubey & S.E.Freire | bristle | 0.79 (5)* | 0.16 (2) | 3.38 (5)* |  |
| Gnaphalieae | *Gamochaeta erythractis* | (Wedd.) Cabrera | bristle | 0.8 (1) | 0.28 (1) | 2 (1) |  |
| Gnaphalieae | *Gamochaeta lulioana* | S.E.Freire & Iharl. | bristle | 0.68 (3) | 0.26 (2) | 2.5 (2) |  |
| Gnaphalieae | *Luciliocline burkartii* | (Cabrera) Anderb. & S.E.Freire | bristle | 0.75 (1)* | 0.24 (1) | 4.25 (1)* |  |
| Gnaphalieae | *Luciliocline longifolia* | (Cuatrec. & Aristeg.) M.O.Dillon & Sagást. | bristle | 1.31 (6)* | 0.4 (5) | 7 (1)* |  |
| Gnaphalieae | *Luciliocline santanica* | (Cabrera) Anderb. & S.E.Freire | bristle | 1.25 (1)* |  | 5.75 (1)* |  |
| Gnaphalieae | *Luciliocline subspicata* | (Wedd.) Anderb. & S.E.Freire | bristle | 1 (1)* |  | 5.75 (1)* |  |
| Gnaphalieae | *Mniodes pulvinata* | Cuatrec. | bristle | 1.09 (2) | 0.19 (1) | 4.05 (2)* |  |
| Gnaphalieae | *Pseudognaphalium lacteum* | (Meyen & Walp.) Anderb. | bristle | 0.6 (3)* |  | 3.03 (3)* |  |
| Gnaphalieae | *Pseudognaphalium meridanum* | (Aristeg.) Anderb. | bristle | 0.55 (2)* | 0.28 (1) | 3.25 (1)* |  |
| Liabeae | *Chrysactinium acaule* | (Kunth) Wedd. | bristle | 2 (1) | 1 (1) | 5 (1)* |  |
| Millerieae | *Aphanactis cocuyensis* | Cuatrec. | epappose | 1.25 (1)* | 0.5 (1) | 0 (1) |  |
| Millerieae | *Aphanactis jamesoniana* | Wedd. | epappose | 1.65 (1)* | 0.55 (1)* | 0 (1) |  |
| Millerieae | *Coespeletia timotensis* | (Cuatrec.) Cuatrec. | epappose | 2.7 (1)* | 0.85 (1)* | 0 (1) |  |
| Millerieae | *Espeletia lopezii* | Cuatrec. | epappose | 4.95 (2)* | 2.05 (1)* | 0 (1) |  |
| Millerieae | *Espeletia pycnophylla* | Cuatrec. | epappose | 2.67 (4) | 1.04 (4) | 0 (1) |  |
| Millerieae | *Espeletiopsis colombiana* | (Cuatrec.) Cuatrec. | epappose | 3.14 (3) | 0.99 (2) | 0 (1) |  |
| Millerieae | *Espeletiopsis pannosa* | (Standl.) Cuatrec. | epappose | 1.5 (1) |  | 0 (1) |  |
| Mutisieae | *Chaetanthera pulvinata* | (Phil.) Hauman | bristle | 2.38 (2)* |  | 5.53 (4)* |  |
| Mutisieae | *Chaetanthera revoluta* | (Phil.) Cabrera | bristle | 2.95 (2)* |  | 3.53 (3)* |  |
| Mutisieae | *Chaptalia cordata* | Hieron. | bristle | 3.67 (3)* | 0.55 (2) | 6.64 (3) |  |
| Mutisieae | *Oriastrum stuebelii* | (Hieron.) A.M.R.Davies | bristle | 4.8 (1) |  | 5.5 (1)* |  |
| Mutisieae | *Trichocline reptans* | (Wedd.) Hieron. | bristle | 6.94 (2) | 4.16 (1) | 11 (1) |  |
| Nassauvieae | *Perezia ciliosa* | (Phil.) Reiche | bristle | 2.84 (2)* | 0.81 (1) | 7.5 (1)* |  |
| Nassauvieae | *Perezia coerulescens* | Wedd. | bristle | 3 (3)* | 1.23 (2)* | 14.17 (3)* |  |
| Nassauvieae | *Perezia multiflora* | (Humb. & Bonpl.) Less | bristle | 4.56 (10)* | 2.41 (9) | 8.48 (9)* |  |
| Nassauvieae | *Perezia pungens* | (Humb. & Bonpl.) Less. | bristle | 4.1 (1) | 1 (1) | 16 (1)* |  |
| Senecioneae | *Chersodoma jodopappa* | (Sch.Bip.) Cabrera | bristle | 2.4 (2) | 0.7 (1) | 7.4 (3)* |  |
| Senecioneae | *Culcitium canescens* | Bonpl. | bristle | 1.73 (2) | 0.61 (1) | 12.11 (2)* |  |
| Senecioneae | *Culcitium nivale* | Kunth | bristle | 2.45 (1)* | 0.6 (1)* | 6.8 (1) |  |
| Senecioneae | *Dorobaea pimpinellifolia* | (Kunth) B.Nord. | bristle | 3.18 (5) | 0.55 (5) | 6.04 (5) |  |
| Senecioneae | *Gynoxys cuicochensis* | Cuatrec. | bristle | 3.25 (1)* | 0.8 (1)* | 5.4 (1) |  |
| Senecioneae | *Gynoxys miniphylla* | Cuatrec. | bristle |  |  | 5 (1) |  |
| Senecioneae | *Gynoxys tomentosissima* | Cuatrec. | bristle | 1.9 (1)* |  | 8.05 (3)* |  |
| Senecioneae | *Lasiocephalus longipenicillatus* | (Sch.Bip. ex Sandwith) Cuatrec. | bristle | 2.06 (2)* | 0.75 (1) | 8.82 (2)* |  |
| Senecioneae | *Lasiocephalus ovatus* | Willd. ex Schltdl. | bristle | 2.28 (2) | 0.7 (2) | 10.27 (4) |  |
| Senecioneae | *Monticalia andicola*  *(Pentacalia andicola)* | (Turcz.) C.Jeffrey. | bristle | 1.47 (7)* | 0.55 (6) | 3.88 (7)* |  |
| Senecioneae | *Monticalia arbutifolia*  *(Pentacalia arbutifolia)* | (Kunth) C.Jeffrey | bristle | 3.38 (3) | 0.46 (2) | 7.54 (5)* |  |
| Senecioneae | *Monticalia imbricatifolia*  *(Pentacalia imbricatifolia)* | (Sch.Bip. ex Wedd.) C.Jeffrey | bristle | 1.75 (1)* |  | 5.8 (1)* |  |
| Senecioneae | *Monticalia myrsinites* | (Turcz.) C.Jeffrey | bristle | 1.95 (1)* | 0.5 (1)* | 3.9 (1) |  |
| Senecioneae | *Monticalia peruviana* | (Pers.) C.Jeffrey | bristle | 2.96 (4)* | 0.66 (4)* | 7.3 (5) |  |
| Senecioneae | *Monticalia vaccinioides*  *(Pentacalia vaccinioides)* | (Kunth) C.Jeffrey | bristle | 2.1 (2)* | 0.55 (1)* | 4.83 (3)* |  |
| Senecioneae | *Rockhausenia apiculata*  *(Werneria apiculata)* | (Sch.Bip.) D.J.N.Hind | bristle | 3.45 (1)* | 1 (1) | 6.76 (2)* |  |
| Senecioneae | *Rockhausenia cochlearis*  *(Werneria cochlearis)* | (Griseb.) D.J.N.Hind | bristle | 3.3 (1)* | 1 (1)* | 7.35 (1)* |  |
| Senecioneae | *Rockhausenia nubigena*  *(Werneria nubigena)* | (Kunth) D.J.N.Hind | bristle | 3.95 (1)* | 1 (1) | 12.3 (4)* |  |
| Senecioneae | *Rockhausenia orbignyana*  *(Werneria orbignyana)* | (Wedd.) D.J.N.Hind | bristle | 3.1 (1) | 1 (1) | 9.5 (2)* |  |
| Senecioneae | *Rockhausenia pectinata*  *(Werneria pectinata)* | (Lingelsh.) D.J.N.Hind | bristle | 1.95 (1)* | 0.65 (1)* | 5.49 (2)* |  |
| Senecioneae | *Rockhausenia pumila*  *(Werneria pumila)* | (Kunth) D.J.N.Hind | bristle | 2.75 (2)* | 0.73 (2)* | 5.5 (2)* |  |
| Senecioneae | *Rockhausenia pygmaea*  *(Werneria pygmaea)* | (Gillies ex Hook. & Arn.) D.J.N.Hind | bristle | 2.77 (3) | 0.6 (2) | 7.35 (3)* |  |
| Senecioneae | *Rockhausenia villosa*  *(Werneria villosa)* | (A.Gray) D.J.N.Hind | bristle | 3.6 (1)* | 0.95 (1)* | 6.11 (2)* |  |
| Senecioneae | *Senecio adenophyllus* | Walp. | bristle | 2.5 (1) | 0.72 (1) | 7.26 (2)* |  |
| Senecioneae | *Senecio algens* | Wedd. | bristle | 3 (1) | 0.72 (1) | 7.18 (2)* |  |
| Senecioneae | *Senecio anconquijae* f. *discoideus* | Cabrera | bristle | 1.1 (2) | 0.34 (2) | 6.44 (2) | 6.48 (1) |
| Senecioneae | *Senecio apolobambensis* | Cabrera | bristle | 1.8 (1) | 0.7 (1) | 8.3 (1) |  |
| Senecioneae | *Senecio asplenifolius* | Griseb. | bristle | 3 (1) | 0.63 (1) | 6 (1) |  |
| Senecioneae | *Senecio candollei* | Wedd. | bristle | 1 (1) | 0.5 (1) | 7.76 (1) |  |
| Senecioneae | *Senecio chionogeton* | Wedd. | bristle | 1.36 (1) | 0.37 (1) | 10.09 (2) |  |
| Senecioneae | *Senecio formosus* | Kunth | bristle | 2.98 (2)* | 0.7 (1) | 8.03 (2)* |  |
| Senecioneae | *Senecio funckii* | Sch.Bip. ex Wedd. | bristle | 2.98 (3) | 0.43 (3) | 5.3 (3) |  |
| Senecioneae | *Senecio humillimus* | Sch.Bip. | bristle | 1.35 (2) | 0.64 (2) | 3.54 (3) | 4.9 (1) |
| Senecioneae | *Senecio melanandrus* | (Wedd.) J.Calvo, A.Granda & V.A.Funk | bristle | 2.15 (1)* | 0.5 (1) | 5.5 (1)* |  |
| Senecioneae | *Senecio menesesiae* | J.Calvo | bristle | 2.85 (1)* | 0.75 (1)* | 7 (1)* |  |
| Senecioneae | *Senecio neeanus* | Cuatrec. | bristle | 3.91 (2) | 0.54 (2) | 7.42 (2) |  |
| Senecioneae | *Senecio nutans* | Sch.Bip. | bristle | 3.43 (2)* | 0.85 (1) | 4.86 (2)* |  |
| Senecioneae | *Senecio puchei* | Phil. | bristle | 2.5 (1) | 0.58 (1) | 5 (1) |  |
| Senecioneae | *Senecio rufescens* | DC. | bristle | 2.07 (1) | 0.52 (1) | 6 (1) |  |
| Senecioneae | *Senecio scorzonerifolius* | Meyen & Walp. | bristle | 3.5 (1) | 0.73 (1) | 7 (1) |  |
| Senecioneae | *Senecio spinosus* | DC. | bristle | 2.97 (3) | 0.65 (3) | 5 (1) |  |
| Senecioneae | *Senecio tephrosioides* | Turcz. | bristle | 2.04 (6) | 0.52 (6) | 8.06 (5) |  |
| Senecioneae | *Senecio wedglacialis* | Cuatrec. | bristle | 3.05 (1)* | 0.6 (1)* | 9.45 (1)* | 4.12 (1) |
| Senecioneae | *Werneria digitata*  *(Xenophyllum digitatum)* | Wedd. | bristle | 3.7 (1)* | 1.05 (1)* | 7.8 (1)* | 3.96 (1) |
| Senecioneae | *Werneria humilis*  *(Xenophyllum humile)* | Kunth | bristle | 1.54 (3)* | 0.43 (2)* | 5.54 (4)* |  |
| Senecioneae | *Werneria poposa*  *(Xenophyllum paposum)* | Phil. | bristle | 2.43 (3)* | 0.55 (2)* | 4.83 (2)* |  |

Supplementary data Table S2 List of samples used for DNA analysis (81 species). For species in which the herbarium label had a synonym of the name used in this study, original species name as in the label were put in parentheses. SRA = Sequence read archive (Accession number). Herbaria acronyms as follows: LPB = Herbarium La Paz (Bolivia), HQCA = Herbarium Quito – Pontifica Universidad Católica del Ecuador (Ecuador), BC = Herbarium of the Instituto Botánico de Barcelona (Spain), FMB = Herbarium Federico Medem Bogotá, Instituto Alexander von Humboldt (Colombia).

| Tribe | Species name | Herbarium | Collector | N collection | Date | SRA | No. of quality-filtered paired reads | No. of reads on target | Enrichment efficiency (% reads on target) | No. of genes assembled at 50% | Total bp recovered | Efficiency recovery (% bp recovered compared to reference) |
| --- | --- | --- | --- | --- | --- | --- | --- | --- | --- | --- | --- | --- |
| Anthemideae | *Cotula mexicana* (DC.) Cabrera | LPB | Ortuño, T. | 984 | 24/03/2010 | ERR6041629 | 4971076 | 1664393 | 33.5 | 274 | 212016 | 73.7 |
| Astereae | *Blakiella bartsiifolia* (S.F.Blake) Cuatrec. | QCA | Gámez, L. | 1120 | 20/09/2012 | ERR6041616 | 2771622 | 724181 | 26.1 | 298 | 224163 | 77.9 |
| Astereae | *Diplostephium eric*oides (Lam.) Cabrera | QCA | Vargas, O.M. | 488 | 08/08/2012 | ERR6041630 | 3435118 | 740493 | 21.6 | 289 | 220614 | 76.7 |
| Astereae | *Diplostephium glandulosum* Hieron. | QCA | Pedersen, H.B. | 45 | 29/03/1989 | ERR6041632 | 3461313 | 830609 | 24 | 291 | 219264 | 76.2 |
| Astereae | *Erigeron ecuadoriensis* Hieron. | QCA | Muriel, P. | | 01/03/2018 | ERR6041635 | 3396766 | 738958 | 21.8 | 285 | 214755 | 74.7 |
| Astereae | *Erigeron rosulatus* Wedd. | LPB | Beck, St. G. | 32527 | 04/12/2007 | ERR6041636 | 2497995 | 381294 | 15.3 | 266 | 200748 | 69.8 |
| Astereae | *Linochilus floribundus* (Benth.) O.M.Vargas  (*Diplostephium floribundum*) | QCA | Vargas, O. M. | 496 | 10/08/2012 | ERR6041631 | 4144689 | 898030 | 21.7 | 278 | 216741 | 75.4 |
| Astereae | *Linochilus rupestris* (Kunth) O.M.Vargas  (*Diplostephium rupestre*) | QCA | Sklenar, P. |  | 03/05/1995 | ERR6041633 | 4554426 | 1041925 | 22.9 | 268 | 198849 | 69.1 |
| Astereae | *Oritrophium peruvianum* (Lam.) Cuatrec. | LPB | Berg, A. | 5319 | 12/10/1997 | ERR6041656 | 1360479 | 210461 | 15.5 | 241 | 190548 | 66.2 |
| Astereae | *Oritrophium repens* (Kunth) Cuatrec. | QCA | Vargas, O. M. | 462 | 04/08/2012 | ERR6041657 | 1598794 | 243706 | 15.2 | 263 | 207276 | 72.1 |
| Astereae | *Parastrephia lucida* (Meyen) Cabrera | LPB | Beck, St. G. | 30668 | 21/11/2007 | ERR6041617 | 1971945 | 609223 | 30.9 | 202 | 165396 | 57.5 |
|  | (*Parastrephia phyliciformis*) |  |  |  |  |  |  |  |  |  |  |  |
| Astereae | *Parastrephia quadrangularis* (Meyen) Cabrera | LPB | Garcia, C. | 67 | 08/03/2006 | ERR6041658 | 1828831 | 265613 | 14.5 | 262 | 200832 | 69.8 |
| Barnadesieae | *Chuquiraga jussieui* J.F.Gmel. | QCA | Irazabal, R. J. | 309 | 13/04/2013 | ERR6041628 | 6842051 | 1012944 | 14.8 | 220 | 176694 | 61.4 |
| Cichorieae | *Hieracium avilae* Kunth | BC | Christenhusz, M.J.M | CO-II-2017-1 |  | ERR7618429 | 2972818 | 366682 | 12.3 | 255 | 188646 | 65.6 |
| Cichorieae | *Hieracium frigidum* Wedd. | QCA | AguirreAguirre, N. | 48 | 06/09/2012 | ERR6041642 | 3664718 | 616781 | 16.8 | 239 | 182178 | 63.3 |
| Cichorieae | *Hypochaeris echegarayi* Hieron. | LPB | Meneses, R.I. | 4855 | 20/01/2009 | ERR6041643 | 7277373 | 1576753 | 21.7 | 307 | 241914 | 84.1 |
| Cichorieae | *Hypochaeris eremophila* Cabrera | LPB | Beck, St. G. | 30692 | 13/02/2008 | ERR6041644 | 6377627 | 1030635 | 16.2 | 306 | 238452 | 82.9 |
| Cichorieae | *Hypochaeris meyeniana* (Walp.) Griseb. | LPB | Meneses, R.I. | 4919 | 20/03/2009 | ERR6041645 | 5008786 | 864463 | 17.3 | 300 | 234816 | 81.6 |
| Cichorieae | *Hypochaeris mucida* Domke | LPB | Meneses, R.I. | 5003 | 27/03/2010 | ERR6041646 | 4422293 | 887337 | 20.1 | 302 | 233616 | 81.2 |
| Cichorieae | *Hypochaeris sessiliflora* Kunth | QCA | Gámez, L. | 1489 | 22/11/2012 | ERR6041647 | 5043434 | 1121928 | 22.2 | 304 | 231387 | 80.4 |
| Cichorieae | *Hypochaeris taraxacoides* (Meyen & Walp.) Ball | LPB | Vidaurre, P. | 1329 | 13/05/2009 | ERR6041648 | 8640824 | 1926911 | 22.3 | 311 | 239574 | 83.3 |
| Coreopsideae | *Bidens andicola* Kunth | LPB | Beck, St. G. | 35094 | 29/01/2016 | ERR6041626 | 5999103 | 1764544 | 29.4 | 311 | 239202 | 83.2 |
| Eupatorieae | *Ageratina gracilis* (Kunth) R.M.King & H.Rob. | FMB | Diazgranados, M. | 4223 | 06/02/2017 | ERR6041622 | 2503273 | 1110891 | 44.4 | 296 | 224637 | 78.1 |
| Eupatorieae | *Mikania brachyphylla* Hieron. | QCA | Lozano, P | 100 | 12/01/2002 | ERR6041652 | 1919891 | 307109 | 16 | 191 | 154896 | 53.9 |
| Eupatorieae | *Oxylobus glandulifer* (Sch.Bip. ex Hemsl.) A.Gray ex Klatt | QCA | Gámez, L. | 1443 | 17/05/2013 | ERR6041623 | 2990819 | 1425332 | 47.7 | 284 | 215457 | 74.9 |
| Gnaphalieae | *Achyrocline alata* DC. | QCA | Jaramillo, J. | 20965 | 27/02/1999 | ERR6041611 | 3459493 | 1511482 | 43.7 | 266 | 204891 | 71.2 |
| Gnaphalieae | *Andicolea ilinissae* (Benth.) Mayta & Molinari | QCA | Muñoz, L. | 368 | 08/03/1984 | ERR6041650 | 2171978 | 892096 | 41.1 | 265 | 203055 | 70.6 |
| Gnaphalieae | *Andicolea thuyoides* (Lam.) Mayta & Molinari | QCA | Irazabal, R. J. | 1028 | 04/10/2014 | ERR6041613 | 1153571 | 507244 | 44 | 179 | 154155 | 53.6 |
| Gnaphalieae | *Belloa kunthiana* (DC.) Anderb. & S.E.Freire | QCA | Irazabal, R. J. | 223 | 16/01/2013 | ERR6041614 | 3945298 | 1643384 | 41.7 | 282 | 218640 | 76 |
|  | (*Lucilia kunthiana*) |  |  |  |  |  |  |  |  |  |  |  |
| Gnaphalieae | *Belloa radians* (Benth.) Sagást. & M.O.Dillon | QCA | Gámez, L. | 1189 | 20/09/2012 | ERR6041612 | 2737294 | 1066519 | 39 | 265 | 203163 | 70.6 |
| Gnaphalieae | *Belloa schultzii* (Wedd.) Cabrera | LPB | Meneses, R.I. | 5004 | 27/03/2010 | ERR6041625 | 3746839 | 919432 | 24.5 | 281 | 214302 | 74.5 |
| Gnaphalieae | *Chryselium gnaphalioides* (Kunth) Urtubey & S.E.Freire | QCA | Holm-Nielsen, L. | 28790 | 14/11/1980 | ERR6041639 | 3277634 | 868425 | 26.5 | 262 | 202866 | 70.5 |
|  | (*Gnaphalium antennarioides*) |  |  |  |  |  |  |  |  |  |  |  |
| Gnaphalieae | *Gamochaeta lulioana* S.E.Freire & Iharl. | LPB | Alberto, H. | 342 | 28/03/2012 | ERR6041638 | 5236130 | 919638 | 17.6 | 266 | 208047 | 72.3 |
| Gnaphalieae | *Luciliocline santanica* (Cabrera) Anderb. & S.E.Freire | LPB | Urtubey, E. | 492 | 27/03/2010 | ERR6041615 | 2253995 | 1015420 | 45 | 259 | 200775 | 69.8 |
| Gnaphalieae | *Luciliocline subspicata* (Wedd.) Anderb. & S.E.Freire | LPB | Beck, St. G. | 30693 | 13/02/2008 | ERR6041651 | 1441999 | 484742 | 33.6 | 271 | 201582 | 70.1 |
| Gnaphalieae | *Pseudognaphalium lacteum* (Meyen & Walp.) Anderb. | LPB | Beck, St. G. | 30689 | 13/02/2008 | ERR6041640 | 4262148 | 1072032 | 25.2 | 288 | 224076 | 77.9 |
|  | (*Gnaphalium lacteum*) |  |  |  |  |  |  |  |  |  |  |  |
| Liabeae | *Chrysactinium acaule* (Kunth) Wedd. | QCA | Madsen, J.E. | 85615 | 22/11/1989 | ERR6041606 | 3607842 | 769098 | 21.3 | 284 | 213018 | 74.1 |
| Millerieae | *Aphanactis jamesoniana* Wedd. | QCA | Salgado, S. | 1702 | 16/11/2014 | ERR6041621 | 1648892 | 640465 | 38.8 | 297 | 224025 | 77.9 |
| Millerieae | *Coespeletia timotensis* (Cuatrec.) Cuatrec. | QCA | Gámez, L. | 1160 | 20/09/2012 | ERR6041619 | 2224422 | 442515 | 19.9 | 277 | 211485 | 73.5 |
| Millerieae | *Espeletia lopezii* Cuatrec. | FMB | Diazgranados, M. | 4528 | 29/11/2017 | ERR6041618 | 2242637 | 617398 | 27.5 | 262 | 206583 | 71.8 |
| Millerieae | *Espeletia pycnophylla* Cuatrec. | QCA | Ulloa, C. | 2398 | 05/10/2012 | ERR6041637 | 5422210 | 985756 | 18.2 | 282 | 210300 | 73.1 |
|  | (*Espeletia pycnophylla* subsp. *angelensis* |  |  |  |  |  |  |  |  |  |  |  |
| Millerieae | *Espeletiopsis colombiana* (Cuatrec.) Cuatrec. | FMB | Diazgranados, M. | 4214 | 06/02/2017 | ERR6041620 | 3756339 | 743690 | 19.8 | 278 | 209961 | 73 |
| Mutisieae | *Oriastrum stuebelii* (Hieron.) A.M.R.Davies | LPB | Beck, St. G. | 30677 | 21/11/2007 | ERR6041624 | 6335101 | 2640369 | 41.7 | 145 | 130818 | 45.5 |
| Nassauvieae | *Perezia ciliosa* (Phil.) Reiche | LPB | Meneses, R.I. | 4560 | 29/02/2008 | ERR6041661 | 11035880 | 3661592 | 33.2 | 208 | 163782 | 56.9 |
| Nassauvieae | *Perezia coerulescens* Wedd. | LPB | Beck, St. G. | 30678 | 21/11/2007 | ERR6041662 | 7402536 | 3525204 | 47.6 | 188 | 152397 | 53 |
| Nassauvieae | *Perezia multiflora* (Humb. & Bonpl.) Less | LPB | Jimenez, I. | 7247 | 27/01/2016 | ERR6041663 | 8140175 | 1638018 | 20.1 | 209 | 162840 | 56.6 |
| Senecioneae | *Chersodoma jodopappa* (Sch.Bip.) Cabrera | LPB | Pozo, P. | 755 | 25/03/2012 | ERR6041627 | 3145419 | 519567 | 16.5 | 309 | 233487 | 81.2 |
| Senecioneae | *Culcitium canescens* Bonpl. | LPB | Meneses, R.I. | 5445 | 05/04/2012 | ERR6041668 | 2364302 | 450344 | 19 | 280 | 214020 | 74.4 |
|  | (*Senecio canescens*) |  |  |  |  |  |  |  |  |  |  |  |
| Senecioneae | *Culcitium nivale* Kunth | QCA | Duchicela, S. A. | 24 | 16/11/2014 | ERR6041609 | 2065967 | 334837 | 16.2 | 237 | 184440 | 64.1 |
| Senecioneae | *Dorobaea pimpinellifolia* (Kunth) B.Nord. | QCA | Sklenar, P | 13140 | 04/12/2010 | ERR6041634 | 3460378 | 895129 | 25.9 | 274 | 206409 | 71.8 |
| Senecioneae | *Gynoxys cuicochensis* Cuatrec. | QCA | Jorgensen, P. M. | 760 | 22/11/1994 | ERR6041641 | 2076734 | 626023 | 30.1 | 227 | 178026 | 61.9 |
| Senecioneae | *Gynoxys miniphylla* Cuatrec. | QCA | Aguirre, N. | 44 | 13/09/2012 | ERR6041607 | 1909001 | 422505 | 22.1 | 240 | 183690 | 63.9 |
| Senecioneae | *Lasiocephalus longipenicillatus* (Sch.Bip. ex Sandwith) Cuatrec. | QCA | Gámez, L. | 1442 | 25/10/2012 | ERR6041649 | 3580585 | 883045 | 24.7 | 272 | 208755 | 72.6 |
| Senecioneae | *Lasiocephalus ovatus* Willd. Ex Schltdl. | QCA | Duchicela, S. A. | 145 | 16/11/2014 | ERR6041608 | 1420262 | 346335 | 24.4 | 226 | 172809 | 60.1 |
| Senecioneae | *Monticalia arbutifolia* (Kunth) C.Jeffrey | QCA | Romoleroux, K. | 5128 | 20/08/2008 | ERR6041659 | 1808934 | 453992 | 25.1 | 246 | 195966 | 68.1 |
| Senecioneae | *Monticalia imbricatifolia* (Sch.Bip. ex Wedd.) C.Jeffrey | QCA | Gámez, L. | 1481 | 22/11/2012 | ERR6041660 | 7190439 | 2155356 | 30 | 295 | 234351 | 81.5 |
| Senecioneae | *Monticalia myrsinites* (Turcz.) C.Jeffrey | QCA | Aguirre, N. | 69 | 19/09/2012 | ERR6041653 | 1829661 | 533484 | 29.2 | 221 | 176679 | 61.4 |
| Senecioneae | *Monticalia peruviana* (Pers.) C.Jeffrey | QCA | Duchicela, S. A. | 1407 | 16/11/2014 | ERR6041654 | 2247599 | 448541 | 20 | 202 | 167202 | 58.1 |
| Senecioneae | *Monticalia vaccinioides* (Kunth) C.Jeffrey | BC | Maarten J. C | | 01/02/2017 | ERR6041605 | 396960 | 57430 | 14.5 | 90 | 86610 | 30.1 |
| Senecioneae | *Rockhausenia apiculata* (Sch.Bip.) D.J.N.Hind | LPB | Beck, St. G. | 30657 | 21/11/2007 | ERR6041679 | 11059164 | 3891751 | 35.2 | 294 | 224106 | 77.9 |
| Senecioneae | *Rockhausenia nubigena* (Kunth) D.J.N.Hind | QCA | Duchicela, S. A. | 65 | 16/11/2014 | ERR6041680 | 3531374 | 1186092 | 33.6 | 282 | 223512 | 77.7 |
| Senecioneae | *Rockhausenia orbignyana* (Wedd.) D.J.N.Hind | LPB | Beck, St. G. | 35001 | 01/11/2015 | ERR6041681 | 1933473 | 512715 | 26.5 | 246 | 192747 | 67 |
| Senecioneae | *Rockhausenia pumila* (Kunth) D.J.N.Hind | QCA | Irazabal, R. J. | 153 | 18/08/2012 | ERR6041682 | 1550328 | 482139 | 31.1 | 262 | 203940 | 70.9 |
| Senecioneae | *Rockhausenia villosa* (A.Gray) D.J.N.Hind | LPB | Jimenez, I. | 8088 | 03/03/2016 | ERR6041683 | 3419834 | 1145544 | 33.5 | 284 | 213900 | 74.4 |
| Senecioneae | *Senecio adenophyllus* Walp. | LPB | Beck, St. G. | 30566 | 10/03/2006 | ERR6041664 | 6969020 | 1766085 | 25.3 | 298 | 227721 | 79.2 |
| Senecioneae | *Senecio algens* Wedd. | LPB | Beck, St. G. | 4458 | 04/12/2007 | ERR6041665 | 2642635 | 623994 | 23.6 | 287 | 219060 | 76.2 |
| Senecioneae | *Senecio apolobambensis* Cabrera | LPB | Meneses, R.I. | 5015 | 27/03/2010 | ERR6041666 | 2439263 | 520349 | 21.3 | 271 | 209181 | 72.7 |
| Senecioneae | *Senecio candollei* Wedd. | LPB | Meneses, R.I. | 4562 | 29/02/2008 | ERR6041667 | 3108614 | 837589 | 26.9 | 259 | 200697 | 69.8 |
| Senecioneae | *Senecio chionogeton* Wedd. | LPB | Palabral | 421 | 21/07/2006 | ERR6041669 | 4835710 | 788255 | 16.3 | 280 | 212832 | 74 |
| Senecioneae | *Senecio formosus* Kunth | QCA | Gámez, L. | 1466 | 25/10/2012 | ERR6041670 | 4567146 | 1265697 | 27.7 | 275 | 214287 | 74.5 |
| Senecioneae | *Senecio funckii* Sch.Bip. ex Wedd. | QCA | Gámez, L. | 1432 | 25/10/2012 | ERR6041671 | 4581886 | 1555742 | 34 | 278 | 215472 | 74.9 |
| Senecioneae | *Senecio humillimus* Sch.Bip. | LPB | Beck, St. G. | 30669 | 21/11/2007 | ERR6041672 | 7935680 | 2441454 | 30.8 | 281 | 219018 | 76.1 |
| Senecioneae | *Senecio neeanus* Cuatrec. | LPB | Pozo, P. | 796 | 13/03/2012 | ERR6041673 | 5674423 | 967946 | 17.1 | 297 | 228255 | 79.4 |
| Senecioneae | *Senecio nutans* Sch.Bip. | LPB | Pozo, P. | 799 | 26/03/2012 | ERR6041674 | 6517167 | 1497298 | 23 | 311 | 241548 | 84 |
| Senecioneae | *Senecio puchei* Phil. | LPB | Meneses, R.I. | 5422 | 13/03/2012 | ERR6041675 | 7013150 | 2093052 | 29.8 | 297 | 226074 | 78.6 |
| Senecioneae | *Senecio rufescens* DC. | LPB | Meneses, R.I. | 4424 | 04/12/2007 | ERR6041676 | 4561490 | 1379068 | 30.2 | 285 | 221994 | 77.2 |
| Senecioneae | *Senecio spinosus* DC. | LPB | Jimenez, I. | 7251 | 27/01/2016 | ERR6041677 | 5269376 | 1345808 | 25.5 | 292 | 225591 | 78.4 |
| Senecioneae | *Senecio tephrosioides* Turcz. | QCA | Aguirre, N. | 92 | 06/09/2012 | ERR6041678 | 3626633 | 580293 | 16 | 291 | 221787 | 77.1 |
| Senecioneae | *Werneria humilis* Kunth | QCA | Salgado, S. | 1705 | 16/11/2014 | ERR6041610 | 3708854 | 1615902 | 43.6 | 244 | 183933 | 63.9 |
| Senecioneae | *Werneria poposa* Phil. | LPB | Pozo, P. | 752 | 25/03/2012 | ERR6041684 | 4265331 | 1403233 | 32.9 | 288 | 220221 | 76.6 |

Supplementary data Table S 3 Fourth-corner analysis results.

Fourth-corner analysis results using 3,000 permutations (model 3: permuting species data within each row of the site x species L matrix) to test for the significance of the statistic, using Holm correction for multiple comparisons to obtain an adjusted p-value. Significance code are as follows: ‘**’ 0.01.

| Test | Stat | Obs | Std.Obs | Alter | Pvalue | Pvalue.adj |  |
| --- | --- | --- | --- | --- | --- | --- | --- |
| Distance.to.equator / achene.length | r | -0.148 | -2.793 | two-sided | 0.007 | 0.028 | * |
| Elevation / achene.length | r | -0.096 | -1.846 | two-sided | 0.061 | 0.121 |  |
| Minimum T / achene.length | r | 0.152 | 2.915 | two-sided | 0.003 | 0.015 | * |
| Distance.to.equator / PL/AL | r | 0.120 | 2.303 | two-sided | 0.023 | 0.069 | . |
| Elevation / PL/AL | r | 0.184 | 3.483 | two-sided | 0.001 | 0.004 | ** |
| Minimum T / PL/AL | r | -0.195 | -3.698 | two-sided | 0.000 | 0.003 | ** |
| Distance.to.equator / range.size | r | 0.260 | 5.122 | two-sided | 0.000 | 0.003 | ** |
| Elevation / range.size | r | 0.059 | 1.138 | two-sided | 0.250 | 0.250 |  |
| Minimum T / range.size | r | -0.200 | -3.935 | two-sided | 0.000 | 0.003 | ** |

Supplementary data Table S4 Phylogenetic signal for diaspore traits and distribution ranges.

Phylogenetic signal for species diaspore traits and distribution ranges using Pagel’s λ (see methods) to test phylogenetic signal (p<0.01 indicates significant signal).

| Trait | Pagel’s λ | p-value likelihood ratio test | No. species with  data in phylogenetic tree |
| --- | --- | --- | --- |
| Achene length | 0.745 | <0.001 | 80 |
| Pappus length | 0.838 | <0.001 | 81 |
| Pappus-to-achene length ratio | 0.676 | <0.001 | 80 |
| Range size | 0.761 | 0.068 | 81 |
